# Oxidative stress and inflammation in the pathogenesis of neurological disorders: Mechanisms and implications

**DOI:** 10.1016/j.apsb.2024.10.004

**Published:** 2024-10-16

**Authors:** Umesh Chandra Dash, Nitish Kumar Bhol, Sandeep Kumar Swain, Rashmi Rekha Samal, Prabhat Kumar Nayak, Vishakha Raina, Sandeep Kumar Panda, Rout George Kerry, Asim K. Duttaroy, Atala Bihari Jena

**Affiliations:** a School of Biotechnology, Campus 11, Kalinga Institute of Industrial Technology (KIIT) Deemed to be University, Bhubaneswar 751024, Odisha, India; b Post Graduate Department of Biotechnology, Utkal University, Bhubaneswar 751004, Odisha, India; c ICMR-National Institute of Pathology, Sadarjang Hospital Campus, New Delhi 110029, Delhi, India; d CSIR-Institute of Minerals & Materials Technology, Bhubaneswar 751013, Odisha, India; e Bioanalytical Sciences, Research and Development, Enzene Biosciences Limited, Pune 410501, Maharashtra, India; f Department of Nutrition, Institute of Medical Sciences, Faculty of Medicine, University of Oslo, Oslo 0317, Norway; g National Centre for Cell Science, Savitribai Phule Pune University Campus, Pune 411007, India

**Keywords:** Neurodegeneration, Oxidative stress, ROS, Neuroprotection, Mitochondrial dysfunction, Aging

## Abstract

Neuroprotection is a proactive approach to safeguarding the nervous system, including the brain, spinal cord, and peripheral nerves, by preventing or limiting damage to nerve cells and other components. It primarily defends the central nervous system against injury from acute and progressive neurodegenerative disorders. Oxidative stress, an imbalance between the body's natural defense mechanisms and the generation of reactive oxygen species, is crucial in developing neurological disorders. Due to its high metabolic rate and oxygen consumption, the brain is particularly vulnerable to oxidative stress. Excessive ROS damages the essential biomolecules, leading to cellular malfunction and neurodegeneration. Several neurological disorders, including Alzheimer's, Parkinson's, Amyotrophic lateral sclerosis, multiple sclerosis, and ischemic stroke, are associated with oxidative stress. Understanding the impact of oxidative stress in these conditions is crucial for developing new treatment methods. Researchers are exploring using antioxidants and other molecules to mitigate oxidative stress, aiming to prevent or slow down the progression of brain diseases. By understanding the intricate interplay between oxidative stress and neurological disorders, scientists hope to pave the way for innovative therapeutic and preventive approaches, ultimately improving individuals' living standards.

## Graphical abstract

Oxidative stress triggers a catastrophic cascade: mitochondrial dysfunction, neuronal death, neuroinflammation, and neurodegeneration, leading to devastating neurodegenerative diseases, necessitating advanced therapies to halt this destructive process.

**Figure:**
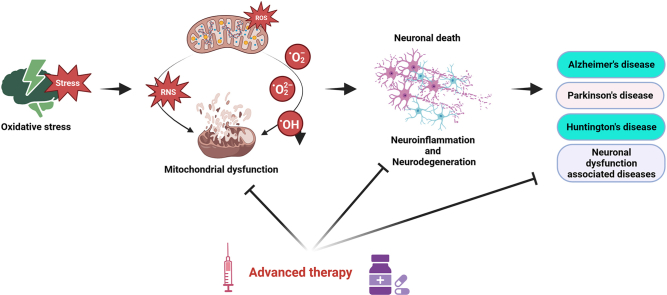

## 1 Introduction

Neuroprotection refers to the body's strategies to protect the nervous system's integrity and functionality, including the brain, spinal cord, and peripheral nerves. This proactive strategy aims to prevent or limit damage to nerve cells (neurons) and other nervous system components, preserving cognitive, sensory, and motor functions. Primarily, it defends the central nervous system (CNS) against injury due to both acute (*e*.*g*., stroke or trauma) and progressive neurodegenerative disorders (*e*.*g*., dementia, Parkinson's, Alzheimer's, epilepsy, etc.)^1^^,^^2^. Herbal medicine and nutraceuticals are crucial and valuable sources for neurological problem prevention rather than treatment. Phytoconstituents have purportedly been proven to have modulatory effects on the nervous system in numerous experimental models of neurological disorders^3^.

**Figure 1:**
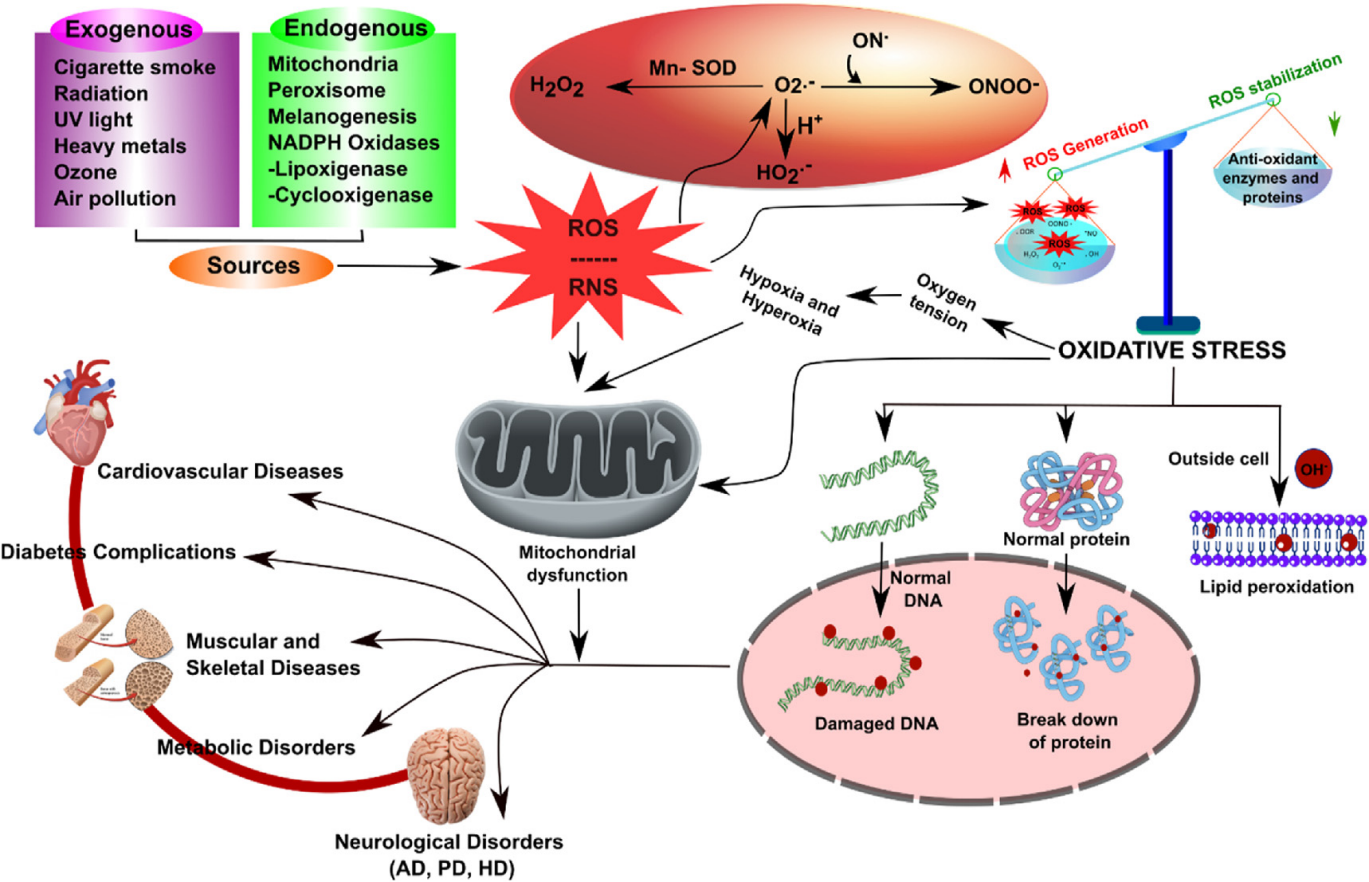
The complex cellular and molecular pathways associated with oxidative stress in various disorders. Key elements include the generation of ROS, cellular damage, and the initiation of signaling pathways that result in significant consequences for triggering the diseases. Additionally, schematic pathways may be imperative for developing personalized therapeutic approaches in the treatment of stress-related oxidative diseases.

Although the pathophysiology of the nervous system is not fully known, most studies on diverse neural disorder models that replicate significant aspects of the disease have identified essential elements such as oxidative stress, mitochondrial dysfunction, neuro-inflammation etc. In neurological diseases and disorders, oxidative stress is an important topic. It refers to an imbalance between the body's natural defense mechanisms' ability to counteract or repair the ensuing damage and the generation of reactive oxygen species (ROS) (Fig. 1). This oxidative stress is crucial in developing neurological disorders that affect the brain and nervous system. Because of its high metabolic rate and oxygen consumption, the brain is an organ that is particularly vulnerable to oxidative stress. Excess ROS, which includes molecules such as superoxide radicals, hydroxyl radicals, and hydrogen peroxide, can damage essential biomolecules such as proteins, lipids, and DNA^4^ (Fig. 1). This triggers a chain of events that leads to cellular malfunction and, eventually, leads to neurodegeneration. A variety of neurological illnesses, including Alzheimer's disease (AD), Parkinson's disease (PD), amyotrophic lateral sclerosis (ALS), Huntington's disease (HD), multiple sclerosis (MS), and ischemic stroke, are linked to oxidative stress. Understanding the impact of oxidative stress on these disorders is critical to develop new treatment methods. Researchers are looking at using antioxidants and other molecules that can help protect against oxidative stress and prevent or reduce the progression of diseases in the brain^5^. Scientists hope to discover new paths for therapy and prevention by better understanding the complicated interplay between oxidative stress and neurological disorders, thereby enhancing the quality of life for people affected by these conditions. Understanding the impact of oxidative stress in these disorders is critical to developing new treatment methods. Researchers are looking at using antioxidants and other molecules that can help protect against oxidative stress and prevent or reduce the progression of diseases in the brain. Scientists hope to discover new paths for therapy and prevention by better understanding the complicated interplay between oxidative stress and neurological disorders, thereby enhancing the quality of life for people affected by these conditions^6^.

## 2 Oxidative stress mechanisms

### 2.1 Molecular and cellular response to oxidative stress

**Figure 2:**
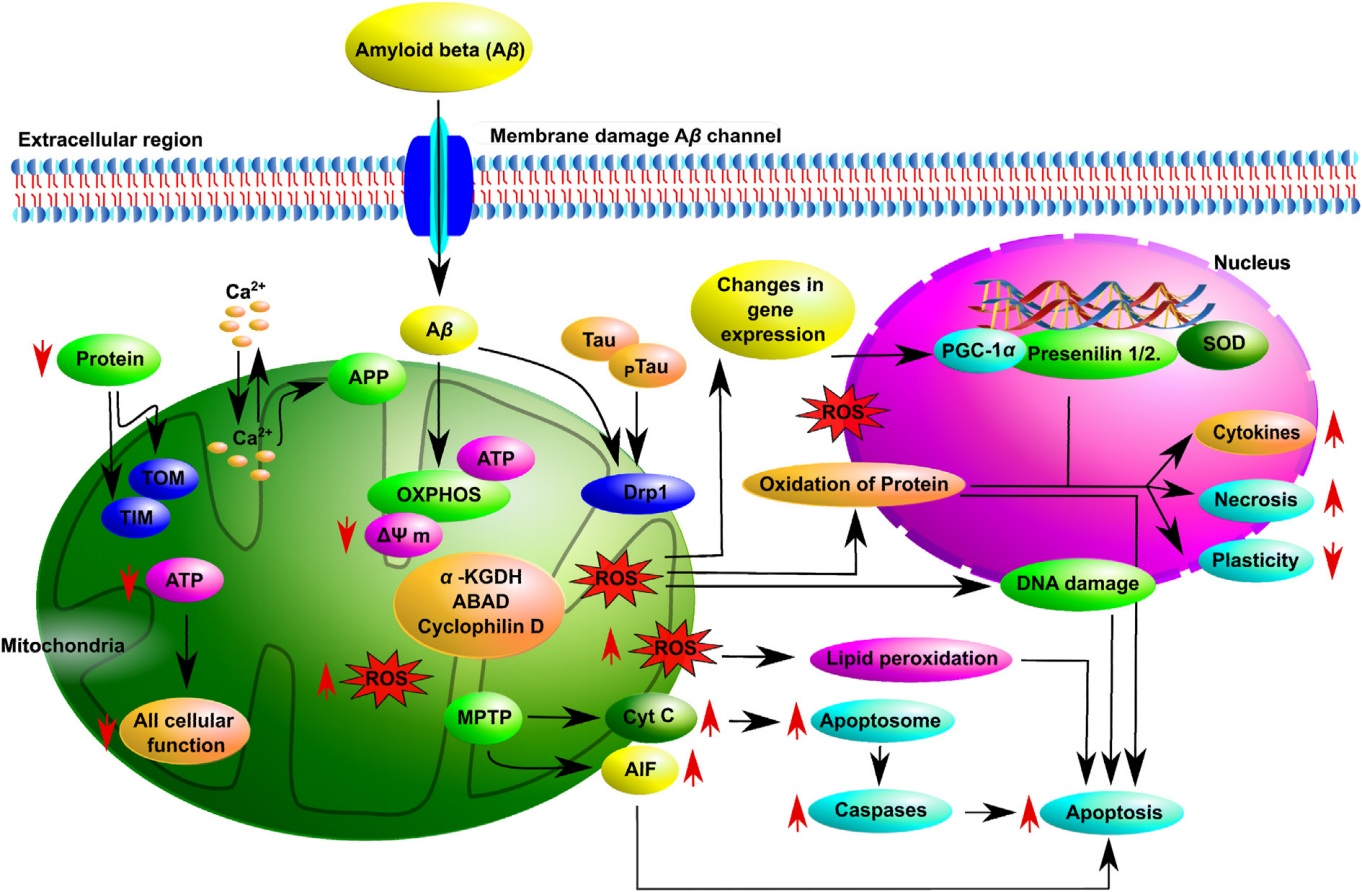
The schematic representation illustrates the complexities of redox-regulated mitochondrial dysfunctions and their mechanisms in neurodegenerative diseases. The figure also depicts the generation of ROS in diverse pathways, eventually resulting in the oxidation of proteins within the nucleus and the reduction of proteins in the mitochondria.

Whether internal or external, stress disrupts an organism's internal balance (homeostasis) and impacts cells at various levels, including structure, function, stability, growth, and survival. Stressors like ionizing radiation, oxygen level changes, chemotherapy drugs, and senescence trigger ROS responses at the cellular level, activating antioxidant mechanisms and signaling pathways^7^^,^^8^ (Fig. 1). Excessive stress can trigger this response into a death signal, leading to apoptosis, necrosis (Fig. 2), or cancer due to genomic instability^9^.

Cell responses to stress are specific to species and cell type and affect critical functions such as cell cycle regulation, protein repair, chromatin stability, damaged protein removal, and metabolism. Understanding these responses is crucial for overall organism health^10^^,^^11^. Eukaryotic cells respond diversely to oxygen fluctuations, affecting growth and function. Normal oxygen levels (21% O_2_) support optimal enzymatic processes and physiological balance. Hypoxic (5%–10% O_2_) or hypertoxic (60%–95% O_2_ with 0.5% CO_2_) conditions trigger complex mammalian responses, including adjustments in breathing, cardiovascular (CV) functions, and specific tissue reactions (angiogenesis, erythropoiesis, glycolysis)^12^^,^^13^ (Fig. 1). Hypoxia enhances anaerobic glycolysis, alters lipid metabolism, and damages membranes, posing survival challenges. Chronic hypoxia alters cellular metabolism and gene expression, while severe hyperoxia induces cell death. Moderate hyperoxia (*e*.*g*., 70% oxygen) inhibits cell growth, induces apoptosis, and alters gene expression^14^. Oxygen-rich environments reduce cellular metabolism, whereas hypoxia activates HIF-1 to maintain ATP levels by adjusting utilization rates^15^. Both hypoxia and hyperoxia trigger signaling events *via* mitochondrial-derived oxygen species (Fig. 1), disrupting metabolic equilibrium. Despite challenges, they find application in therapies for hemorrhagic shock, nerve regeneration, and ischemia-reperfusion injury. Hypoxia shows promise in treating obesity by suppressing appetite at high altitudes^16^. Understanding cellular responses to stress and oxygen fluctuations is critical for advancing therapies and improving health outcomes across diverse conditions.

### 2.2 The vicious cycle of ROS, reactive nitrogen species (RNS), and mitochondrial dysfunction in oxidative stress

Oxidative stress impacts cells through mitochondria, which generate 90% of cellular energy as ATP and regulate calcium metabolism and thermogenesis. Dysfunctional mitochondria produce high levels of ROS and RNS, leading to oxidative damage in DNA, proteins, and lipids (Fig. 1). ATP production begins with the breakdown of glucose into pyruvic acid, transforming into NADH and acetyl CoA and involves three enzyme classes^17^. Mitochondria generates ROS, including superoxide anion, hydrogen peroxide, and hydroxyl radicals, managed by enzymes like Mn-SOD and Cu/Zn-SOD (Fig. 1). Peroxisomes also contribute to ROS production and contain catalase to decompose hydrogen peroxide. Oxidative stress arises from an imbalance of ROS/RNS, leading to cellular damage and diseases like diabetes, CV issues, and cancer. Antioxidant systems like glutathione S transferase and thioredoxin (Trx) neutralize excessive ROS/RNS, but persistent oxidative stress can contribute to aging and cancer^18^^,^^19^. ROS also mediates physiological responses such as cell differentiation, proliferation, and migration^20^.

Oxidative stress involves nitric oxide (NO) production, resulting from an imbalance in ROS/RNS regulation. NO has diverse roles, including neuronal signaling and blood pressure regulation, but excessive NO can harm cells^21^, influence apoptosis, and trigger inflammation. NO can induce cytochrome *c* release from mitochondria, activating the caspase-dependent apoptotic pathway and binding to cytochrome *c* oxidase, leading to mitochondrial dysfunction. NO synthesis by nitric oxide synthases (NOS) from L-arginine involves NOS isoforms, including nNOS, eNOS, iNOS, and mtNOS^22^. NO's interaction with mitochondrial superoxide generates peroxynitrite, contributing to diseases like stroke, heart disease, diabetes, inflammation, cancer, neurodegenerative disorders, and aging^22^.

### 2.3 Oxidative stressors and their impact on human health

#### 2.3.1 Age and oxidative stress

**Figure 3:**
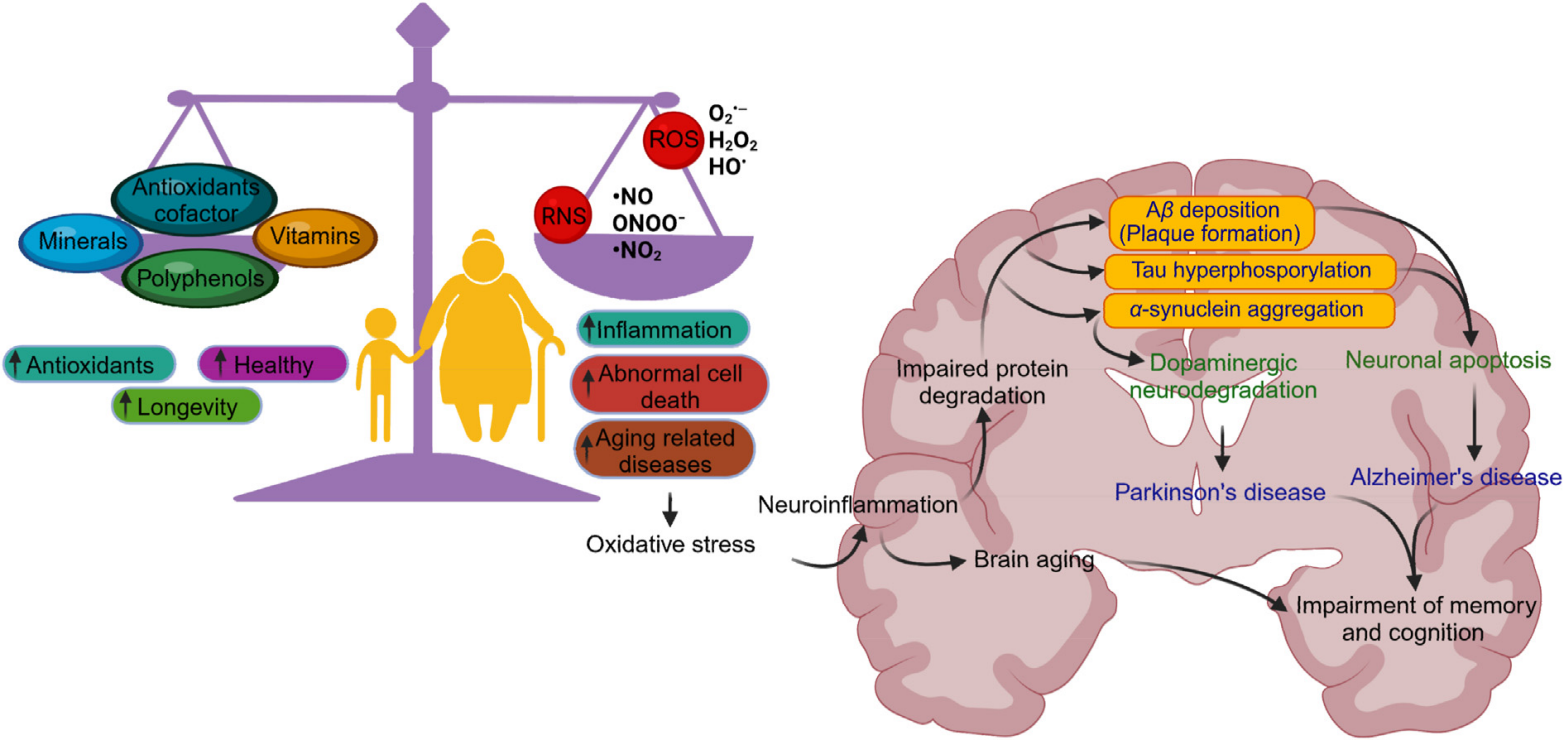
The correlation between aging, oxidative stress, and neurodegenerative diseases. It illustrates the involvement of RNS and ROS in initiating cell death, concurrently contributing to aging-related diseases. Furthermore, the diagram also depicts the compensatory role of therapeutic agents (minerals, vitamins, and antioxidants) in mitigating age-associated diseases by combating oxidative stress.

Aging is the gradual decline in tissue and organ functionality over time^23^. The oxidative stress theory of aging posits that this decline results from accumulating oxidative damage to macromolecules like lipids, DNA, and proteins caused by reactive oxygen and nitrogen species (RONS)^24^ (Fig. 3). The precise molecular mechanisms underlying oxidative stress-induced aging are not fully understood. Still, heightened levels of RONS are thought to trigger cellular senescence, a protective response that halts cellular proliferation in the face of replication-associated damage. Senescent cells adopt an irreversible senescence-associated secretory phenotype (SASP), releasing soluble factors such as interleukins, chemokines, and growth factors^25^^,^^26^. They also secrete degradative enzymes like matrix metalloproteases (MMPs) and insoluble proteins/extracellular matrix (ECM) components. The induction of cellular senescence by RONS involves interaction with various elements of the SASP^25^^,^^26^, which are implicated in age-related disorders and oxidative stress through modulation of mammalian target of rapamycin (mTOR) complex functions.

*Interleukin-1 alpha (IL-1α)* expression initiates a cascade leading to a proinflammatory state that activates nuclear factor-kappa-B (NF-*κ*B) and promotes epithelial–mesenchymal transition, thereby facilitating tumor metastasis. Increased MMP expression, observed in conditions like cancer, AD, atherosclerosis, osteoarthritis, and lung emphysema, is linked to age-related and chronic disorders. FOXO proteins, critical in the insulin/insulin-like growth factor-1 (IGF 1) mediated defense against oxidative stress, are inhibited, contributing to increased RONS levels. Reduced sarco/endoplasmic reticulum Ca^2+^ ATPase activity plays a role in cardiac senescence onset. Inhibition of sirtuins leads to elevated RONS levels due to decreased superoxide dismutase (SOD) activity, promoting a pro-inflammatory state that impairs sirtuin-mediated inhibition of tumor necrosis factor-alpha (TNF*α*) and NF-*κ*B. Sirtuin inhibition also prevents the suppression of *c-Jun* and *c-Myc*, contributing to tumorigenesis. The p16INK4a/pRB and p53/p21 signaling pathways tightly regulate cellular senescence^25^.

Age-related disease due to oxidative stress: Oxidative stress, an imbalance between ROS and antioxidant defenses, plays a crucial role in various physiological and pathological processes. One such process is cellular senescence, a state of irreversible growth arrest that can be induced by oxidative stress. As a consequence of cellular senescence, cells secrete various SASP factors. These SASP factors have been implicated in the development and progression of several acute and chronic pathological conditions, including cardiovascular diseases (CVDs), acute and chronic kidney disease (CKD), neurodegenerative diseases (NDDs), macular degeneration (MD), biliary diseases, and cancer. CV risk factors, such as obesity, diabetes, hypertension, and atherosclerosis, are linked to the activation of the inflammatory pathway, which is mediated by the cytokines IL-1*α*, IL-6, and IL-8^7^.

Additionally, an increase in cellular senescence has been observed among these risk factors. Additionally, it should be noted that vascular calcification is intricately associated with a process known as SASP-driven osteoblastic transdifferentiation, which involves transforming senescent smooth muscle cells into osteoblast-like cells. In numerous neurodegenerative disorders, such as AD, examinations of brain tissue biopsies reveal elevated quantities of p16, MMP, and IL-6^8^. Chronic obstructive pulmonary disease, biliary cirrhosis, cholangitis, and osteoarthritis exhibit overlapping detrimental SASP profiles, characterized by the presence of interleukin-6 (IL-6), interleukin-8 (IL-8), and matrix metalloproteinase (MMP). The initiation of epithelial to mesenchymal transition (EMT) facilitated by RONS enhances the process of cancer metastasis. A theoretical framework known as the oxidation-inflammatory theory of ageing takes into account the complex interactions between oxidative stress, inflammation, and the ageing process. Aging is characterized by the gradual deterioration of homeostasis, primarily attributed to the persistent presence of oxidative stress. This oxidative stress predominantly impacts the regulatory mechanisms, including the nervous, endocrine, and immune systems. The subsequent initiation of the immune system triggers an inflammatory response that establishes a feedback loop wherein chronic oxidative stress and inflammation mutually reinforce one another, thereby leading to heightened age-associated morbidity and mortality^9^^,^^10^.

#### 2.3.2 Endogenous and exogenous stressors in ROS generation

Free radicals are produced due to exogenous stressors such as pollution, cigarette smoke, or internal processes (endogenous) when antioxidant defenses are overwhelmed. Environmental triggers like cigarette smoke, UV radiation, heavy metal ions, ozone, allergens, drugs, toxins, pollutants, pesticides, or insecticides can all elevate the production of ROS in cells^11^^,^^12^ (Fig. 1). Ionizing radiation converts radicals into organic hydroperoxides and hydrogen peroxide. Alpha particle exposure intensifies oxygen levels and hastens peroxide production in fibroblasts^13^^,^^14^. Ultraviolet radiation (UVA) induces oxidative reactions, generating 8-oxo-guanine and depleting intracellular glutathione (GSH)^15^. Heavy metals like iron, copper, cadmium, nickel, arsenic, and lead induce free radicals through chemical reactions, affecting cellular components and DNA. Ozone exposure causes inflammation in the respiratory epithelium, impacting lung function, even in healthy individuals. These factors collectively contribute to cellular oxidative stress^16^^,^^17^.

The primary internal sources of cellular redox-reactive species, encompassing ROS and RNS, include various cellular components like the mitochondrial electron transport chain (ETC), endoplasmic reticulum (ER), peroxisomes, membrane-bound NADPH oxidase (NOX) isoforms 1–5, dual oxidases (Duox) 1 and 2 complexes, and NO synthases isoforms 1–5 (NOS1–5). Mitochondrial ETC complexes I and III are significant producers of superoxide anions. Dysregulated ROS signaling can contribute to various diseases linked to oxidative stress. Cells can also actively produce ROS at low levels for signaling pathways that regulate cell survival, proliferation, and defense mechanisms against invaders. Specific enzymatic systems like the NOX family are dedicated to physiological superoxide radical production. ROS and RNS influence signaling pathways, including nuclear factor-kappa B (NF-*κ*B) activation and translocation. Oxidized NF-*κ*B has reduced DNA binding potential but can be countered by factors like TR or redox factor 1^18^^,^^19^. This interaction affects NF-*κ*B dependent inflammatory signals. Cyclopentenones, electrophilic anti-inflammatory prostaglandins, counter ROS-mediated NF-*κ*B signaling by binding to ROS-modified peptides and proteins. Additionally, endogenous stress, shaped by cellular conditions and gene expression patterns, leads to DNA damage, including double-stranded DNA breaks, often observed in human tumors. Non-enzymatic reactions within the mitochondrial respiratory chain also contribute to these processes^20^^,^^21^.

#### 2.3.3 ROS linked chronic diseases

Endogenous ROS, generated during aerobic metabolism, typically serves as secondary messengers in cell signaling, impacting processes like proliferation, differentiation, and apoptosis. Elevated ROS levels, mislocalized production, or faulty forms can contribute to chronic degenerative diseases due to biomacromolecule damage. Oxidative stress, arising from an imbalance between oxidative and antioxidative processes, is pivotal in developing such conditions^22^^,^^23^. Hypertension and hypercholesterolemia, key CV risk factors, heighten ROS production, causing oxidative stress. Of these factors, hypertension significantly contributes to CV disease development. ROS plays a dual role: low levels offer benefits like anti-atherosclerotic effects, while high levels lead to cell damage, contributing to conditions like endothelial dysfunction, atherosclerosis, and heart-related issues^24^, ^25^, ^26^. Oxidative stress alters gene expression, affecting transcription factors like NF-*κ*B, AP-1, and PPAR, further impacting CVDs^24^. Cancer cells experience constant oxidative stress due to mitochondrial dysfunction and metabolic alterations^27^^,^^28^. They evade cell death by activating oncogenes, like NRF2, which safeguards them from ROS and DNA damage. ROS plays a role in cancer progression, promoting various cellular processes despite causing mutagenesis. In cancer, ROS leads to protein and lipid oxidation, toxic protein carbonyl formation, and the accumulation of cytotoxic products like 4-hydroxy-2-non-enal^29^^,^^30^.

**Figure 4:**
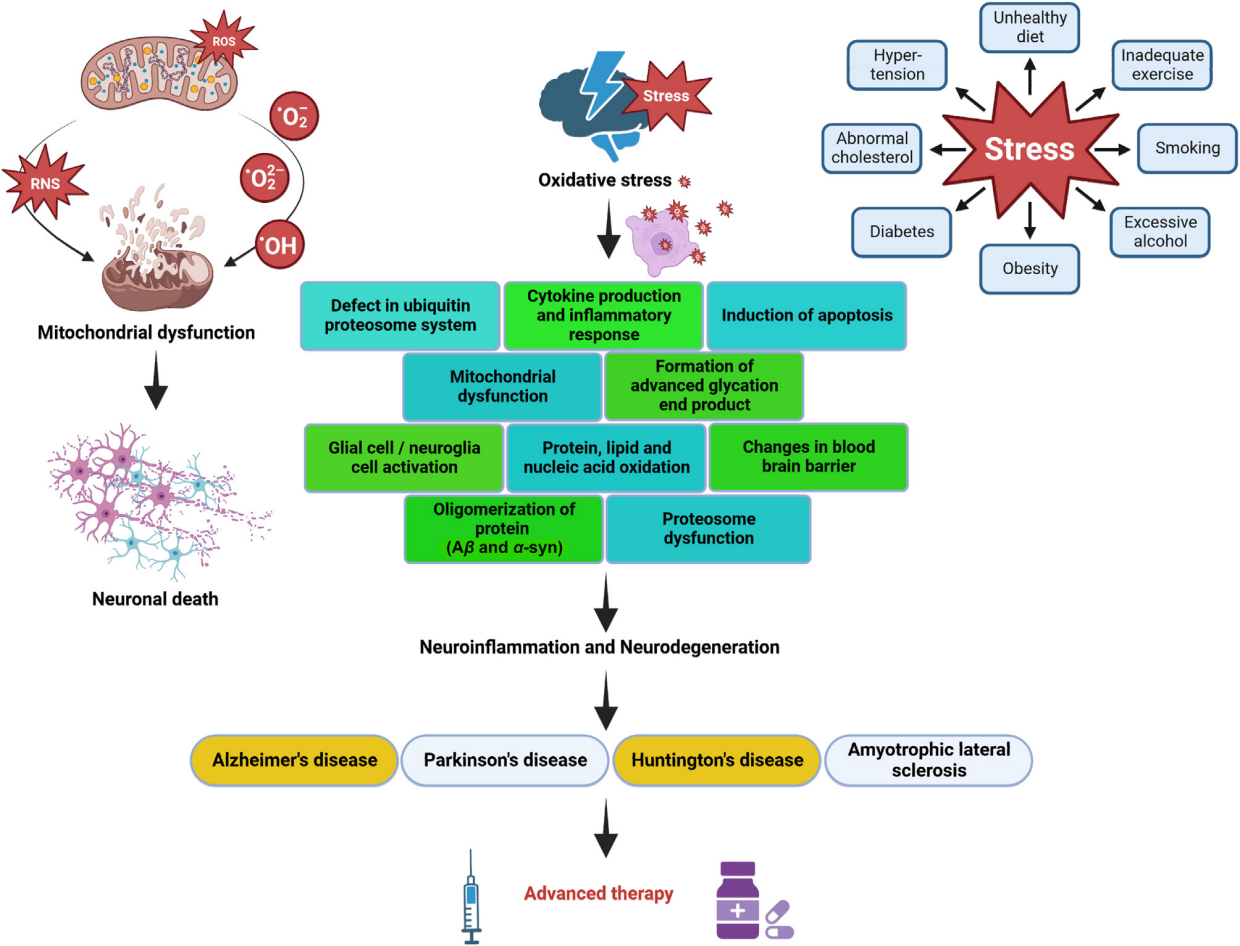
This figure depicts the key relationship between neurological diseases and oxidative stress. It delineates diseases linked to stress, emphasizing the subsequent cascade where oxidative stress becomes a pivotal factor leading to neurodegradation.

Oxidative stresses are not equally sensitive for all brain neurons. Neurons with longer axons and multiple synapses, demanding more energy for functions, are vulnerable when mitochondria are dysfunctional^31^^,^^32^. Dopaminergic neurons face additional oxidative stress due to dopamine metabolism. Common neurodegenerative disorders include AD, PD, HD, ALS, and Friedreich's ataxia, often associated with aging-related mitochondrial DNA (mtDNA) mutations, calcium dysregulation, and ETC decline^33^, ^34^, ^35^. Oxidized DNA, proteins, and lipids in post-mortem neurodegenerative brain tissue highlight oxidative stress's role. Defective metal use by mutant proteins due to oxidative stress is another cause of neurodegeneration^36^. The “free radical theory of aging” proposed over 60 years ago, suggested that ROS causes cellular and tissue damage, leading to aging and age-related diseases (Fig. 3). Aging can result from genetic and external factors, like diet, exercise, drug use, inflammation, smoking, and alcohol. Today, oxidative stress remains a central principle in aging theories, with mitochondria and NOX as key systems generating excessive oxidative stress (Fig. 4). During aging, cells accumulate high-molecular protein aggregates, primarily composed of oxidized proteins and lipids^18^^,^^37^^,^^38^. Maintaining protein homeostasis involves degrading these aggregates. The popularity of antioxidant supplements emerged from the free radical theory, but recent data suggest that they may not significantly reduce age-related disease incidence.

### 2.4 Oxidative stress and neurodegeneration

Cognitive impairment and dementia significantly impact the quality of life and life expectancy among elderly people with neurodegenerative disorders like AD, PD, Huntington's disease (HD), ALS, and vascular dementia exerting profound effects, marked by memory decline, motor impairments, and loss of mobility. Oxidative stress plays a crucial role in the pathophysiology of dementia, with studies demonstrating correlations between oxidative stress biomarkers like malondialdehyde (MDA), glutathione peroxidase (GSH-Px), protein carbonyls (PC), and cognitive decline, particularly in elderly individuals. Elevated GSH-Px activity has been associated with slower cognitive impairment progression. At the same time, increased GSH levels may paradoxically hasten decline, possibly due to heightened oxidative stress and impaired GSH-Px activity. Furthermore, oxidative stress contributes to forming stress granules (SGs), disrupting neuronal function by sequestering essential proteins and transcripts. In NDDs, oxidative stress is complicatedly linked with DNA damage, aberrant protein folding, and aggregation, contributing to tissue damage and disease progression. The brain is vulnerable to oxidative damage heightened by its high metabolic activity and susceptibility to neuroinflammatory responses, originated by activated microglia in response to oxidative damage. Neuroinflammation further exacerbates tissue damage and contributes to disease progression, highlighting oxidative stress as a pivotal factor in NDDs like AD, PD, HD, and ALS diseases.

#### 2.4.1 Role of oxidative stress in coma

Coma is a syndrome characterized by partial loss of consciousness or prolonged profound sleep of the brain and body. Coma is defined in medical science as a state in which the brain loses its attentiveness, and the body ceases to respond to any internal or external stimulation and ceases to experience necessities. If this state persists for an extended period or until death, the patient is said to be in a coma. Coma is a sickness that can affect anyone at any time and in any place. It is becoming more common as a result of high blood pressure caused by contemporary lifestyles, diabetes, brain tumors, nutritional deficiency, hormonal imbalance, toxins, drug addiction, and continuous stress^39^^,^^40^.

The relation between coma and oxidative stress arises from the complex interaction between the physiological condition of unconsciousness and the biochemical consequences of oxidative damage in the brain. Coma can result from various underlying causes, some of which might generate oxidative stress as a secondary consequence. Factors such as traumatic brain traumas or infections, for example, can increase the generation of ROS in the brain, resulting in cellular damage and worsening the severity of the coma. In addition, coma-related processes such as ischemia and inflammation may trigger an environment conducive to oxidative stress, as reduced blood circulation and inflammatory reactions can promote the generation of ROS^41^. As a result, medical solutions for coma care usually include oxidative stress mitigation, such as the use of antioxidant medications and methodologies to reduce oxidative damage. This is extremely important in protecting neural cells and maintaining brain function, mainly when the underlying disorder contains factors that can aggravate oxidative stress^41^^,^^42^.

#### 2.4.2 Role of oxidative stress in Friedreich ataxia (FA)

Friedreich ataxia (FA) is a progressive inherited autosomal recessive genetic neurodegenerative disorder that affects both children and adults. Typically, within a span of 10–20 years, symptoms of ataxia become evident, and following their onset, individuals often find themselves confined to a wheelchair. In the advanced stages of the disease, some individuals may experience complete disability. FA can significantly reduce life expectancy, often due to heart-related complications. However, individuals with milder forms of FA may survive into their 60s^43^. The condition is characterized by symptoms such as ataxia, gait abnormalities, loss of sensory perception, and areflexia. In 96% of FA cases, affected individuals are homozygous for a pathological GAA trinucleotide repeat in intron one of the frataxin genes (*FXN*) located on chromosome 9. Frataxin (FXN) is a crucial protein for the assembly of iron-sulfur cluster-containing enzymes and the production of ATP within the mitochondria. FA results in the degeneration of nerve fibers in the spinal cord and peripheral nerves, causing them to become thinner and leading to complications in the heart, spine, and, in some cases, diabetes. Significantly, FA does not impact an individual's cognitive abilities. While rare, FA represents the most prevalent form of hereditary ataxia worldwide, affecting approximately 1 in every 50,000 people. It is inheritable in both male and female children^44^^,^^45^.

As discussed, FA is predominantly caused by a mutation in the *FXN* gene, which results in reduced production of frataxin, a mitochondrial protein required for iron regulation. This deficit causes an accumulation of iron in the mitochondria, which sets off a chain of events that increases oxidative stress. Mitochondria are an important source of ROS and play an important role in energy production. In FA, faulty iron control affects mitochondrial function, producing excess ROS that destroys cellular components such as DNA, proteins, and lipids. The accumulation of oxidative stress is a significant factor in the progressive neurological symptoms seen in FA, such as muscular weakness, ataxia, and heart problems. Understanding the relationship between FA and oxidative stress is critical for identifying prospective therapeutic options that target the disease's underlying biological pathways and mitigate its impact^46^.

#### 2.4.3 Role of oxidative stress in epilepsy

Epilepsy is a common neurological condition characterized by two or more spontaneous seizures or convulsions. This disorder is caused by the temporary generation of excessive electrical impulses in the brain, resulting in tremors or seizures. Epilepsy is generally classified into three types: a) idiopathic epilepsy, which is thought to have a genetic basis and includes conditions like primary generalized childhood-onset absence epilepsy; b) secondary or symptomatic epilepsy, which results from identifiable CNS injuries or disorders like infections, strokes, traumatic brain injuries, or cerebral dysgenesis; and c) cryptogenic epilepsy, where the etiology is unknown. Idiopathic and cryptogenic epilepsy account for roughly 70% of all occurrences, with the other 30% categorized as symptomatic (secondary)^47^^,^^48^.

Extensive research is currently underway to explore the link between epilepsy and oxidative stress. Oxidative stress is characterized by an imbalance in the production of ROS and the body's capacity to repair the damage. Numerous studies have indicated a connection between epilepsy and oxidative stress. Epileptic seizures can lead to an increased production of ROS in the brain, giving rise to oxidative stress. Seizures elevate metabolic activity and energy demands, potentially generating excess ROS. These free radicals can harm cellular components such as DNA, proteins, and lipids, thereby exacerbating neuronal dysfunction in epilepsy.

Conversely, epilepsy can induce oxidative stress. According to some studies, individuals with epilepsy may possess a compromised antioxidant defense system, rendering them more susceptible to the detrimental effects of ROS^49^. Chronic inflammation, often associated with epilepsy, can also contribute to oxidative stress. While our understanding of the relationship between epilepsy and oxidative stress is advancing, the precise nature of this association and its implications for the development and progression of epilepsy remain active subjects of research. It is crucial to acknowledge that oxidative stress is just one facet of the multifaceted mechanisms underlying epilepsy, necessitating further investigation to reveal the full extent of this connection and its potential therapeutic applications^50^.

#### 2.4.4 Role of oxidative stress in brain hemorrhage

A brain hemorrhage is a severe and potentially fatal form of cerebrovascular event that accounts for about 20% of stroke cases caused by vascular anomalies. This condition involves bleeding within the brain caused by a blood artery obstruction or rupture. Many patients report sudden and deep unconsciousness within minutes, which can be fatal if not treated promptly. The brain is contained within the protective boundaries of the skull, and blood leakage within the skull can cause compression and injury to brain regions. Hemorrhagic strokes occur when bleeding in the brain occurs as a result of a ruptured or leaky blood vessel. With a considerable accumulation of blood, the brain's constricted area within the skull might obstruct the free passage of oxygen-rich blood into brain tissue, potentially producing cerebral edema or brain swelling. The pooled blood creates a mass known as a hematoma, and the increased pressure caused by decreased oxygen supply can cause brain cell death. An intense headache, weakness, or paralysis on one side of the face, arm, or leg, trouble swallowing or seeing, coordination loss, speech impairment, changes in awareness, and drowsiness or coma are common symptoms of a brain hemorrhage^51^.

Oxidative stress is associated with various clinical conditions, including brain hemorrhages. In the event of a brain hemorrhage, the damaged brain tissue experiences an increase in oxidative stress levels. This rise in oxidative stress can be attributed to the delivery of iron from the blood into an environment where it is not typically found. When this iron reacts with hydrogen peroxide, a common ROS, it generates highly reactive hydroxyl radicals. These free radicals can cause oxidative damage to nearby brain cells.

Moreover, the inflammatory response triggered by blood in the brain tissue can exacerbate oxidative stress. The release of inflammatory mediators and the infiltration of immune cells can contribute to producing more ROS^52^. Managing oxidative stress effectively in a brain hemorrhage is crucial, as excessive oxidative damage can worsen the injury and impede the healing process. Antioxidants and anti-inflammatory medications are frequently used to mitigate oxidative stress and reduce future damage to the brain due to bleeding. Individuals at risk of brain hemorrhages, particularly those with conditions like high blood pressure or vascular anomalies, should adopt a healthy lifestyle to minimize oxidative stress. This includes maintaining a balanced diet, engaging in regular exercise, and refraining from smoking and excessive alcohol consumption. These lifestyle changes can help reduce the risk of oxidative stress and its associated complications, such as brain hemorrhages^53^ (Fig. 4).

#### 2.4.5 Role of oxidative stress in dystonia

Dystonia is a complex neurological movement disease characterized by uncontrollable and involuntary muscle spasms. Dystonia can present in a variety of forms, affecting a single muscle (called focal dystonia), a group of muscles (called segmental dystonia), or the entire body (called general dystonia). Dystonia is caused by improper functioning of the basal ganglia, the deep part of the brain responsible for coordinating movements between the brain and other body parts, including muscles, joints, and limbs. The basal ganglion regulates the precise muscle contractions essential for smooth body movements^54^. If this part of the brain is injured, it can result in improper or superfluous muscle contractions during movement. While some dystonia cases may be caused by brain traumas that impair the basal ganglia, many cases have no clear clarification, which makes them idiopathic. Dystonia is a disorder that affects people of all ages and races, ranging from young children to elderly people. Dystonia patients may experience involuntary twisting and odd postures and positions. This illness can affect any body part, including the arms, legs, trunk, face, and vocal cords, yet it is not fatal^55^.

The potential for oxidative stress to contribute to the development and evolution of this complex neurological condition is at the root of the connection between oxidative stress and dystonia. Dystonia, characterized by uncontrollable muscular contractions, is thought to be caused by basal ganglia dysfunction, a deep brain region that helps regulate movement coordination. Oxidative stress, caused by an imbalance between the body's ability to combat ROS and antioxidants, can damage the basal ganglia and disturb its regular functioning. Furthermore, oxidative stress is frequently related to inflammation, which may play a role in the development of dystonia. While further research is needed to understand this relationship completely, it shows that oxidative stress may play a role in the pathogenesis of dystonia and may pave the way for future antioxidant-based treatment strategies^56^.

#### 2.4.6 Role of oxidative stress in dementia and Alzheimer's disease

Dementia is not a specific ailment but rather a collective term encompassing a range of disorders that impact a patient's cognitive functions, communication abilities (understanding and expression of language), memory, and behavior. This includes noticeable shifts in speech, conduct, and cognition, often prompting inquiries into the root causes of these transformations. Various theories have been posited to explain potential triggers for dementia, such as thyroid and parathyroid disorders, diabetes, exposure to toxic chemicals, and heavy metal toxicity. Moreover, certain reversible conditions, like thyroid problems and vitamin deficiencies, can manifest symptoms resembling dementia. Researchers have identified numerous likely contributors to the onset of dementia. AD and vascular dementia together make up the majority, constituting over 80% of dementia cases. The remaining 20% can be attributed to conditions like Creutzfeldt-Jakob disease, Lewy body disease, subcortical leukoencephalopathy, and HD. While a definitive cure remains elusive, early detection can potentially reduce the progression of dementia^57^.

Oxidative stress is becoming increasingly considered a crucial element in the development of dementia, particularly AD. This type of cognitive impairment is characterized by abnormal protein accumulation and brain cell degeneration. It raises the imbalance of oxidative markers and triggers the overproduction of ROS. Elevated ROS levels can disrupt brain cell structures and cause inflammation, contributing to neurodegenerative disorders. Researchers have discovered a relationship between oxidative stress and the production of *β*-amyloid plaques and neurofibrillary tangles, both of which are hallmarks of AD^58^. As a result, the link between oxidative stress and dementia has received significant research, indicating the importance of oxidative stress mitigation measures in dementia prevention and therapy^59^.

Alzheimer's disease (AD) is one of the most well-known and prevalent NDDs, and it is characterized primarily by cognitive decline, memory impairment, and neuronal damage^60^. The substantial body of evidence supports the hypothesis that neuronal injury resulting from an aberrant equilibrium in oxidative stress is likely a pivotal factor in the development and progression of AD^61^, ^62^, ^63^. The previous studies point out a clear connection between oxidative stress and damage to various biomolecules, including protein oxidation, DNA/RNA oxidation, and increased levels of byproducts of lipid peroxidation^61^^,^^64^. However, increased lipid peroxidation levels under oxidative stress are strongly associated with neurotoxicity in AD, leading to an increase in amyloidogenesis through up-regulation of *β*-secretase expression^63^. Since, oxidative stress is a central contributor to AD also impacts mitochondrial function, amyloid-beta (A*β*) production, and overall disease pathogenesis^65^. The mitochondrial cascade hypothesis suggests that AD arises from the gradual buildup of oxidative damage in mtDNA, RNA, lipids, and proteins due to compromised ETC activity^66^. Therefore, mitochondrial dysfunction in AD includes malfunctioning complexes, particularly ATP synthase, a critical player in oxidative phosphorylation, and damage to the promoter region of the mitochondrial ATP synthase gene responsible for ATP generation^67^. Oxidative species notably affect ATP synthase, a significant source of endogenous free-radical production within mitochondria^66^. Lipid peroxidation, a hallmark of AD-related oxidative stress, generates reactive aldehydes like 4-hydroxynonenal (HNE), which modify proteins, including ATP synthase^68^^,^^69^. In AD patients, excessive HNE modification of ATP synthase subunit *α* occurs predominantly in the hippocampus and inferior parietal lobule, leading to reduced enzymatic activity^70^. Dysfunctional mitochondria in AD generate 4-HNE, promoting *γ*-secretase complex upregulation and amyloid precursor protein (APP) cleavage, leading to A*β* accumulation, the major hallmark of AD^71^^,^^72^. Mutant APP cellular lines exhibit altered mRNA and protein levels of mitochondrial fission and fusion proteins^71^. However, mitochondrial dysfunction is independent of intramitochondrial A*β*.

Nevertheless, A*β* deposition in the brain leads to neuroinflammation and oxidative stress by activating microglia, which releases the superoxide radical (Fig. 3). Intracellular buildup of A*β* and phosphorylated tau leads to excessive mitochondrial fragmentation and altered mitochondrial dynamics. Both A*β* and phosphorylated tau increase the GTPase activity of Dynamin-related protein 1 (Drp1), leading to mitochondrial fragmentation. However, A*β* fosters calcium transfer from the endoplasmic reticulum to mitochondria, while tau hinders calcium efflux. The interaction of A*β* with astrocytic calcium-sensing receptors downregulates them, prompting A*β*, NO, and peroxynitrite secretion by neurons, further driving AD pathology. Elevated calcium ions (Ca^2+^) and ROS foster toxic p-tau aggregates^73^, ^74^, ^75^. ROS activate stress kinases, notably the p-JNK pathway, contributing to tau hyperphosphorylation and cell death^76^. This early alteration in mitochondrial function reduces functionality and increased ROS production and contributes to broader issues, including altered lipid metabolism, lipid peroxidation (Fig. 1), and bioenergetic defects. These factors collectively worsen mitochondrial dysfunction and impair effective ER-mitochondrial interaction in AD. Furthermore, the research also highlights a diminished presence of antioxidants in AD patients, encompassing reduced levels of vitamins C and E and decreased concentrations of uric acid and key antioxidant defense enzymes such as catalase and SOD^77^. This insufficiency in antioxidants can significantly intensify oxidative stress within AD.

#### 2.4.7 Role of oxidative stress in Parkinson's disease

Parkinson's disease (PD) is indeed one of the most common NDDs after AD, characterized by both motor and non-motor symptoms. It is a chronic and progressive neurological disorder characterized by the degeneration of dopaminergic neurons in the substantia nigra region of the brain^78^. This degeneration leads to a shortage of dopamine, a neurotransmitter crucial in motor control and other brain functions^79^. Scientific findings indicate that ROS and oxidative stress play crucial roles in the development of PD. Although the precise mechanisms underlying PD remain unclear, research suggests that individuals with PD, particularly in the substantia nigra of the brain, have elevated levels of oxidized proteins, DNA, and lipids, while experiencing reduced levels of the antioxidant GSH.

Moreover, RNS, including high levels of NO generated by NOS near dopaminergic neurons, contribute to nitrosative stress^80^. Importantly, NO inhibits various enzymes within mitochondrial ETC complexes I and IV, thereby increasing ROS levels^81^. Hence, oxidative stress is a substantial contributor to PD. ROS are generated from various sources, including the ETC, external factors, dopamine auto-oxidation, the metabolism of dopamine by monoamine oxidase B, inflammatory reactions, and the presence of heavy metals, all of which collectively contribute to the production of ROS in the context of PD.

Moreover, the progressive buildup of neuromelanin in substantia nigra neurons as a function of age contributes to the generation of ROS and resultant oxidative stress^82^. Furthermore, introducing pesticides and neurotoxins also triggers elevated ROS production, a phenomenon prominently observed in the context of PD^83^. Mitochondrial dysfunction and inflammation are also the key factors contributing to the excessive accumulation of ROS in PD, along with age, elevated iron and calcium levels, and dopamine degradation (Fig. 1).

Moreover, mutations in proteins involved in mitochondrial Complex I function have been linked to PD, resulting in increased ROS production and susceptibility to oxidative stress^84^. This leads to atypical mitochondrial shapes characterized by elongation and fragmentation. Deficiencies in mitochondrial fusion, the interplay between mutant Leucine-rich repeat kinase 2 (*LRRK2*) and *Drp1* that promotes mitochondrial fission, and the adherence of *α*-synuclein to the outer mitochondrial membrane all foster mitochondrial dysfunction in PD^43^^,^^85^. Since mitochondria are also involved in immune and inflammatory responses, functioning as signaling hubs, in PD, inflammatory occurrences are evident, marked by activated microglia, inflammatory macrophages, and elevated proinflammatory cytokine levels^86^^,^^87^.

Consequently, immune system dysregulation, including changes in T cell profiles and heightened NLRP3 inflammasome activation, is linked to PD and might impact disease progression^88^. However, oxidative stress is intricately associated with the aggregation of *α*-synuclein, a key feature of PD. The pathology of disease revolves around the buildup and clustering of *α*-synuclein, a neuronal protein located at presynaptic sites. Oxidative stress is also linked to the creation of Lewy bodies, which consist of aggregated *α*-synuclein and are characteristic protein aggregates identified in the brains of PD patients^89^^,^^90^. Thus, in PD patients, the increased ROS production is due to the substantia nigra exhibiting heightened basal lipid peroxidation and disrupted GSH metabolism, resulting in a deficiency of antioxidant systems^78^.

#### 2.4.8 Role of oxidative stress in Huntington's disease

Huntington's disease (HD) is another neurodegenerative disorder that stems from a mutation in the Huntingtin (HTT) gene, yet the precise mechanisms and HD development remain elusive. Nevertheless, mounting evidence points to heightened oxidative stress in HD patients^80^^,^^91^^,^^92^. This review delves into the role of oxidative stress in HD pathogenesis, exploring mediators and potential mechanisms related to mutant *HTT*-triggered oxidative stress and progression. Multiple pathways contribute to oxidative stress in HD, including elevated NOX activity, oxidation of mitochondrial enzymes, disturbance of active vitamin B6, and activation of the antioxidant defense system^93^^,^^94^. However, mitochondrial dysfunction plays a crucial role in the pathogenesis of HD^94^. mtDNA is crucial in regulating respiratory chain complex activities and contributes to striatal degeneration in HD. HD brains exhibit mitochondrial anomalies, including loss and altered morphogenesis characterized by increased fission and decreased fusion^95^. Notably, the reduction in mtDNA copy number and mtDNA loss are also implicated in the neurodegenerative progression of HD, with specific mutations in the mitochondrial displacement loop (D-loop) of mtDNA associated with an increased HD risk^96^^,^^97^. Thus, elevated levels of oxidative stress are observed in both HD patients and asymptomatic carriers of the HD gene, underscoring its critical role in HD pathogenesis. An early event in the progression of HD is dysfunction in mitochondrial axonal transport, occurring even before the formation of mutant *HTT* aggregates^98^.

Interestingly, a study investigating oxidized proteins in the striatum of HD patients discovered significant carbonylation of the ATP synthase subunit *α* compared to age-matched controls^99^^,^^100^. Furthermore, mitochondrial fractions extracted from the striatum of HD patients exhibited notably reduced ATP synthase catalytic activity compared to controls^101^. While PD and HD share the common feature of oxidative stress, limited observations have explored oxidative modifications targeting ATP synthase in these disorders.

Additionally, cardiomyocytes derived from bacterial artificial chromosome (BAC) mice expressing full-length mutant human *HTT* (BACHD mice) displayed cellular electro-mechanical issues, including extended action potentials, irregular contractions, and relaxation disturbances^102^. These cellular rhythm abnormalities were linked to heightened calcium waves and increased Ca^2+^/calmodulin-dependent protein kinase II (CaMKII) activity, indicating disruptions in calcium balance^103^. Mitochondrial dysfunction can lead to the overproduction of ROS/RNS or the failure of antioxidant defenses, resulting in an oxidative/nitrative stress condition closely linked to HD^104^. This increased oxidative stress was associated with the overexpression of mitochondrial superoxide dismutase 2 (SOD2) and the NEIL3 gene encoding DNA glycosylase^105^. Thus, the imbalances in antioxidant defense enzymes, particularly SOD and GSH-Px activities, were identified in HD, confirming the presence of oxidative stress. Earlier case studies performed meta-analysis comprising 12 studies involving 375 HD patients and 447 healthy controls, the results showed significant increases in blood lipid peroxidation products, 8-hydroxyguanosine levels, and GSH-Px activity in HD patients compared to controls^106^. Conversely, reduced GSH levels were lower in HD patients than in controls. However, no significant differences between HD cases and controls were observed in blood SOD, cholesterol, high-density lipoproteins, and low-density lipoproteins, and triglycerides^105^^,^^106^.

#### 2.4.9 Role of oxidative stress in amyotrophic lateral sclerosis (ALS)

Concisely, ALS is a debilitating disease characterized by intricate mechanisms involving diverse cell types, neuroinflammation, oxidative stress, and mitochondrial dysfunction^107^. Dysregulated mitochondria, essential for energy production and cell survival, are linked directly to ALS pathogenesis^108^. Furthermore, oxidative stress, a prominent disease feature, results in lipid peroxidation, protein damage, and RNA and DNA oxidation in ALS patients. In a study involving 167 ALS patients and 62 age-matched healthy controls, ALS patients exhibited notably elevated plasma lipid peroxidation levels compared to the control group^109^. The previous case study also utilized ALS mouse models and suggested that neurodegeneration is driven by oxidative stress, compromised mitochondria, elevated intracellular calcium levels, inflammation, and the loss of trophic support^93^^,^^108^.

Furthermore, changes in mitochondrial constituents, encompassing respiratory complexes I and III, signified mitochondrial dysfunction, resulting in escalated production of free radicals^110^. Thus, ROS disrupts calcium balance, induces mtDNA mutations, modifies membrane permeability, and fosters lipid oxidation and protein carbonylation. These processes collectively contribute to the onset and progression of ALS. Consequently, the biomarkers of oxidative stress, including MDA-modified proteins and lipid peroxidation products, have been identified in urine, blood, cerebrospinal fluid, and various tissues of ALS patients^111^. The other biomarkers like plasma creatinine, showed significant correlations with plasma uric acid, and urinary oxidative stress biomarkers.

Moreover, indications of oxidative stress were detected in the frontal cortex, implying its early implication in ALS pathology^112^. The observed protein oxidative damage was strongly associated with changes in fatty acid concentrations, particularly those of the *n*-3 series like docosahexaenoic acid^113^. Earlier, the findings revealed that an excessive generation of ROS in conjunction with an ineffective antioxidant defense system represents a notable pathological hallmark in ALS^114^. This is supported by the elevated activity of erythrocyte Cu, Zn-superoxide dismutase (Cu, Zn-SOD) observed in ALS patients, indicative of heightened oxidative stress^115^. In addition to ALS pathology, it was also observed that microglia become chronically activated, transforming a resting to an inflammatory phenotype^116^. While microglia initially respond to neuronal injury in an attempt to provide neuroprotection, their chronic activation in ALS contributes to the ongoing neuroinflammation and neurodegeneration observed in the disease^117^ (Fig. 4). However, oxidative stress and microglial activation are intricately linked in the pathogenesis of NDDs. A deeper understanding of this interplay can provide valuable insights into developing effective therapeutic strategies.

## 3 Mitochondrial dysfunction in neurodegenerative diseases (NDDs)

Neurodegenerative disorders are complex conditions with diverse symptoms and brain region-specific effects. Mitochondrial dysfunction is a standard feature, serving as a convergence point for various pathological pathways^118^^,^^119^. Mitochondria are essential organelles responsible for energy production, cell death regulation, and different cellular processes. Dysfunctional mitochondria contribute to neurodegeneration through increased ROS production and oxidative damage. Both apoptosis and excitotoxicity are major causes of neuronal cell death, with mitochondria playing a pivotal role in both processes^120^^,^^121^ (Fig. 2). Elevated ROS levels can impact mitochondrial functions, including ATP production, membrane potential, permeability transition pore (MPTP) activation, and calcium regulation, ultimately leading to neuronal damage^118^^,^^122^. Evidence of mitochondrial involvement in NDDs emerged with the discovery of complex I deficiency in PD patients' substantia nigra and platelet mitochondria. Subsequent research found deficiencies in other ETC complexes in AD and HD (Fig. 4). Biochemical analysis of postmortem AD brains revealed impaired citric acid cycle enzyme function, correlated with clinical status, and linked to reduced brain metabolism^121^.

In NDDs, mitochondrial dysfunction is a significant concern. A*β* disrupts the integrity of the cell membrane, leading to impaired mitochondrial function. A*β* inhibits the oxidative phosphorylation (OXPHOS) system, resulting in reduced ATP production and increased formation of ROS (Fig. 2). This decline in ATP production affects numerous cellular processes. Additionally, the decrease in mitochondrial membrane potential (Δ*Ψ*_m_) prompts the opening of mitochondrial permeability transition pores (MPTPs). Consequently, cytochrome *c* (cyt *c*) and other proapoptotic factors are released from the intermembrane space, triggering apoptosome formation and caspase activation, ultimately leading to apoptosis^123^ (Fig. 2). Furthermore, cytochrome *c* plays a role in initiating apoptosis by activating apoptotic protease activating factor 1 (Apaf1). Apaf1 assembles into a structure known as the apoptosome within the cytoplasm. The apoptosome, in turn, activates procaspase-9, which is an initiator caspase that is cleaved into its active form, caspase-9 (Fig. 2). Caspase-9 then activates executioner caspases, such as caspase-3, which facilitate the fragmentation of nuclear DNA and the breakdown of the cytoskeleton and nuclear lamina. This process leads to the transformation of cells into a spherical apoptotic structure. Mitochondrial membranes contain Bcl-2 proteins that can either promote or inhibit cell death through interactions with other proteins. Pro-apoptotic proteins like Bax and Bad, for example, facilitate the opening of the mitochondrial permeability transition pore (mPTP). In contrast, anti-apoptotic proteins such as Bcl-2 and Bcl-xL prevent cell death by binding to and blocking the actions of pro-apoptotic proteins. Another proapoptotic factor, apoptosis-inducing factor (AIF), is released by mitochondria in the presence of A*β* AIF translocates to the nucleus and induces apoptosis independently of caspases^124^^,^^125^ (Fig. 2). Furthermore, phosphorylated tau protein (pTau) and^126^ A*β* promote increased nitrosylation of dynamin-related protein-1 (Drp1), impairing mitochondrial dynamics, elevated mitochondrial fission, and neurodegeneration^127^. Additionally, A*β* disrupts protein import into mitochondria and reduces the activity of mitochondrial proteins like amyloid-beta binding alcohol dehydrogenase (ABAD), *α*-ketoglutarate dehydrogenase complex (*α*KGDH), and cyclophilin D (Fig. 2). A*β* and A*β* precursor protein (APP) interfere with mitochondrial calcium ion (Ca^2+^) regulation, causing mitochondrial calcium overload. This, in turn, leads to reduced Δ*Ψ*m, MPTP opening, proapoptotic factor release, increased ROS production, and decreased ATP production (Fig. 2).

## 4 Role of microglia in oxidative stress-induced damage in neurodegenerative diseases (NDDs)

Among neuroglial cells, the two primary types of macroglia, *i*.*e*., oligodendrocytes and astrocytes are the most abundant in the CNS. Oligodendrocytes primarily function to form the lipid-rich myelin sheath, which insulates axons and facilitates rapid transmission of electrical signals. In contrast, astrocytes, a diverse and heterogeneous group, perform numerous critical roles in cerebral development and homeostasis. These roles include maintaining the blood–brain barrier, and astrocytes are essential for both the structural and functional integrity of the brain^128^.

Microglia, the primary immune cells in the CNS, play crucial roles in maintaining CNS balance, responding to chronic stress, and influencing synaptic plasticity, learning, and memory^117^. Notably, microglial cells, the central nervous principal immunocytes of the system, play a vital role in shielding neurons from bacterial and toxic harm^129^. Microglia can either worsen chronic stress and AD damage by dysregulated phagocytosis, releasing inflammatory cytokines, and mediating oxidative stress, or they can help restore neuronal balance. Microglia further plays a role in AD by contributing to A*β* accumulation, tau pathology, neurodegeneration, and synaptic loss^130^. However, they also have beneficial functions, like A*β* clearance^131^. Apart from A*β* accumulation, microglia-induced oxidative stress can activate the NOD-like receptor pyrin domain-containing 3 (NLRP3) inflammasome, leading to the release of mature interleukin-1 beta (IL-1*β*) and interleukin-18 (IL-18)^132^. This process amplifies neuroinflammation and oxidative damage in NDDs. In other NDDs like HD, activated microglia and reactive astrocytes worsen the HD condition by boosting pro-inflammatory genes, leading to persistent inflammation^133^ (Fig. 4). Moreover, in the HD brain, neuroinflammation is evident through reactive morphological in these glial cells^134^. Recent discoveries also highlight the potential of activated microglia and astrocytes to perform essential functions that protect tissue and maintain neuronal function in the HD brain^135^. Elevated oxidative stress in microglia has been linked to a heightened risk of neuronal injury. ROS-mediated stress has been shown to impact the immune function of microglial cells in both inflammatory responses and neurodegenerative disorders^136^. However, activated microglia upregulate the inducible nitric oxide synthase (iNOS) and nicotinamide adenine dinucleotide phosphate (NADPH) oxidase enzymes, generating ROS and RNS^137^. These molecules cause oxidative damage to biomolecules, including lipids, proteins, and nucleic acids, within the nearby microenvironment. This review explores the connection between oxidative stress and NDDs, delves into microglial roles in these diseases, particularly their response to oxidative stress, and highlights recently identified neuroprotective microglial states.

Oligodendrocytes are vulnerable to oxidative stress, which can lead to their death and subsequent demyelination, a hallmark of various neurological diseases, including neurodegenerative disorders. Promising research reveals that oligodendrocytes and their progenitors are involved in the onset and progression of NDDs. These cells are primarily involved in age-related NDDs, starting with multiple system atrophy, which is characterized by oligodendroglia pathology and is linked to AD and PD, traditionally considered neuron-centric. A deeper understanding of oligodendrocyte functions and dysfunctions could pave the way for new disease-modifying treatments for NDDs^93^^,^^138^. Likewise, astrocytes are essential for CNS health and function, playing a significant role in NDDs. They can promote remyelination or exacerbate myelin disruption through inflammatory responses in demyelinating diseases. Astrocytes' dysfunction, including the formation of intracellular aggregates, disrupts their normal functions and harms neuronal health, contributing to diseases such as, HD, PD, AD, and ALS ^93^^,^^138^.

### 4.1 Microglial activation and response to oxidative stress impact disease progression

Microglia is pivotal in the intricate relationship between oxidative stress and NDDs. While their activation can promote neuroinflammation and oxidative harm, recent findings suggest the existence of protective microglial states^129^. A better grasp of the molecular mechanisms controlling microglial responses to oxidative stress may unlock tailored therapeutic approaches for neurodegenerative disorders. However, microglia activation involves distinct cell surface receptor expression, polarization responses, and the release of inflammatory mediators. The activation states are broadly categorized as M1-like (pro-inflammatory and neurotoxic) or M2-like (anti-inflammatory and neuroprotective)^139^. Under conditions of heightened oxidative stress, microglia undergoes phenotypic shifts towards a pro-inflammatory state^140^. This activated microglial state entails the secretion of pro-inflammatory cytokines, notably TNF-*α* and IL-1*β*. M1-like microglia are induced by IFN*γ* and LPS, signaling through various pathways like TLR4 and JAK/STAT^139^^,^^141^. Thus, activated M1-like microglia upregulate pro-inflammatory cell surface markers like MHCII and CD86^142^. Moreover, they stimulate the production of various pro-inflammatory mediators, including cytokines such as TNF*α* and interleukins (IL-1*β*, IL-6, IL-12, IL-17, IL-18, IL-23), chemokines like CCL12 and CXCL10, as well as other pro-inflammatory agents, including ROS and RNS, inducible iNOS, and cyclooxygenase-2 (COX-2)^143^. While M1-like microglia play a role in combating pathogens, chronic activation in diseases contributes to neuroinflammation, oxidative stress, and neurotoxicity. In Myelin Sheath, astrocytes and oligodendrocytes are damaged due to inflammatory lesions and aberrant immune responses, highlighting crucial crosstalk between these cell types in white matter disorders. New therapeutic approaches are focusing on targeting reactive astrocytes in a range of CNS disorders. Natural and synthetic inhibitors show promise for traumatic brain injury, while MAO-B inhibitors and A2A receptor antagonists are potential treatments for AD. Innovative treatments, such as synthetic nanoparticles for spinal cord injury, underscore the potential of astrocyte-focused interventions^144^, ^145^, ^146^.

Moreover, activated microglia also discharge ROS and RNS, thereby contributing to an augmented burden of oxidative injury upon neighboring neuronal and glial cells^136^. Altering microglial responses *via* metabolic reprogramming holds promise for treating AD and other NDDs. Still, additional research is required to understand better the specific connections between metabolic changes and microglial functions.

### 4.2 Signaling pathways involved in microglial activation

Both AD and HD involve the activation of the NF-*κ*B signaling pathway in microglia^147^. In AD, NF-*κ*B is activated in response to A*β* aggregates and contributes to the production of pro-inflammatory cytokines^148^. In HD, mutant huntingtin (mHTT) protein interacts with the I*κ*B kinase (IKK) complex, leading to NF-*κ*B activation and the expression of pro-inflammatory cytokines^149^. NF-*κ*B activation is a central feature of microglial activation in both diseases. Other vital pathways, like the kynurenine pathway, which metabolizes l-tryptophan into neuroactive metabolites, are implicated in microglial activation in AD and HD^150^. Dysregulation of this pathway leads to neurotoxic metabolites like quinolinic acid and 3-hydroxykynurenine^151^. Elevated levels of these metabolites have been observed in both diseases, suggesting their role in neuroinflammation and neurotoxicity. However, dysregulation of the complement system, including the classical complement pathway, is implicated in microglial activation in AD and HD^152^. In AD, complement activation is associated with A*β* plaque clearance and inflammation. In HD, complement components may also play a role in neuroinflammation and phagocytosis of cellular debris. Particularly in AD and PD, MAPK signaling pathways, including the extracellular signal-regulated kinase (ERK), c-Jun N-terminal kinase (JNK), and p38 MAPK pathways, are activated in response to A*β* and other inflammatory stimuli in microglia^153^^,^^154^. These pathways regulate the expression of pro-inflammatory genes and cytokines, contributing to neuroinflammation. Pathological conditions in the CNS activate intracellular signaling pathways, polarizing astrocytes into A1 or A2 phenotypes regulated by pathways like NF-*κ*B, TLRs, MAPK, S1PR, JAK/STAT3, and PI3K/AKT. The mechanisms behind these alterations are still unknown. Astrocyte damage results in demyelination and oligodendrocyte death in conditions such as Alexander disease and vanishing white matter. In osmotic demyelination syndrome, astrocyte death precedes oligodendrocyte loss^144^^,^^145^.

Furthermore, the triggering receptor expressed on myeloid cells 2 (TREM2) is a cell surface receptor expressed on microglia. Mutations in TREM2 are associated with an increased risk of ALS. TREM2 signaling is involved in microglial phagocytosis and regulation of the immune response^155^. While these pathways show commonalities in their involvement in microglial activation, it is important to note that the specific triggers and downstream consequences may differ between these NDDs due to the distinct pathological features of each disease.

## 5 Cellular mechanism and antioxidant defense to overcome the neurodegenerative disorder

Antioxidants exert their influence on the process of autoxidation through the disruption of free radical propagation or the inhibition of free radical formation, employing various mechanisms. These compounds can scavenge the species responsible for initiating peroxidation, disrupting the autoxidative chain reaction, extinguishing the O_2_^•−^ radicals, and impeding the generation of peroxides^156^. Antioxidants that can impede the free radical cascade represent the most productive agents in combating oxidative stress. Phenolic or aromatic rings are present within these compounds, thereby enabling the antioxidants to facilitate the donation of hydrogen radicals (·OH) to the free radicals that arise because of oxidation. The stabilization of the radical intermediate occurs through the resonance delocalization of the electron within the aromatic ring^157^.

The pivotal function of antioxidants lies in their ability to effectively halt oxidative chain reactions by eliminating free radical intermediates, as elucidated by^158^. Numerous investigations have consistently demonstrated that the cellular redox status holds paramount importance in the context of ROS-mediated signaling and mitochondrial function^159^. The significant reduction in intracellular GSH levels leads to a pronounced increase in the generation of ROS within the mitochondria, consequently initiating the depolarization of the mitochondrial membrane^160^. As elucidated, the fundamental aspect of inducing antioxidant defense enzymes and modulating intracellular GSH in response to stress lies in the stimulation of the Nrf2/ARE pathway^161^. The reversal of GSH depletion and restoration of transcriptional activity associated with antioxidant response elements (AREs) to their basal levels can be achieved through the administration of *N*-acetylcysteine, as demonstrated by Limón-Pacheco et al.^162^ in 2007. Maintaining optimal intracellular ROS levels is paramount in facilitating physiological redox signaling. This process involves activating and regulating endogenous defense mechanisms, safeguarding cells against the detrimental effects of nitrosative, oxidative, and electrophilic stress^163^. Supplementation with exogenous antioxidants has diminished the enhancements in insulin sensitivity and antioxidant gene expression typically observed following exercise^164^. This highlights the significance of endogenous antioxidant enzymes induced by ROS in restoring the body's physiological redox balance.

Moreover, the upregulation of thioredoxin (Trx) has been experimentally shown to impede the advancement of insulin resistance *in vivo*, encompassing both type 1 and type 2 diabetes, as reported by Yamamoto et al.^165^ in 2008. The latest discoveries indicate that Nrf2 exhibits a safeguarding effect against oxidative stress in aging, as de Oliveira et al.^166^ reported in 2018. The diminishment of Nrf2 functionality has been ascertained to be a contributing factor in the pathogenesis of age-related disorders such as AD and PD^167^.

As elucidated by Tan et al.^168^, numerous investigations have unveiled the potential of antioxidant-laden nutrient sources in mitigating the harmful effects of oxidative stress and non-communicable diseases (NCDs) linked to obesity. Undoubtedly, an exceptional amalgamation of bioactive elements can bestow safeguard against the pernicious effects of oxidative stress, a known instigator of inflammatory responses. In substantiation of this claim, a multitude of epidemiological investigations, such as the European paradox study^169^, the WHO/MONICA study^170^, the NHS study^171^, and the Harvard HPSF, have demonstrated an inverse correlation between the presence of antioxidants and the incidence of numerous NCDs, notably CV ailments. The attention of scholars in academia and industry has been captivated by the antioxidant capacity found in natural products to prevent age-related diseases.

Mitochondria-targeted antioxidants have great potential against the damage caused by ROS generation. Their ability to confer more excellent protection against oxidative damage has been attributed to their ability to cross the phospholipid bilayer of mitochondria and thus eliminate ROS^172^. In principle, a broad range of antioxidants could be targeted to mitochondria *via* triphenylphosphonium (TPP) moiety conjugation. In particular, ubiquinol (MitoQ) is the best-characterized antioxidant targeted to mitochondria by conjugation to the TPP cation.

## 6 Precision medicine for neurodegenerative diseases

Neurodegenerative diseases (NDDs) are being studied for precision medicine, using genomics and data mining to categorize individuals into subgroups with varying susceptibility to conditions like AD, PD, and ALS. Gene polymorphisms also influence stroke and epilepsy. Modern tools like microarrays and Next generation sequencing (NGS) offer new avenues for treatment, revealing candidate genes and enabling simultaneous screening of genes associated with neurological disorders. Advancements in sequencing, Genome wide association study (GWAS) and NGS provide insights into transcript structure, differential transcription, and noncoding RNA involvement, highlighting potential causative links between differentially methylated regions and neurological disorders^173^.

### 6.1 Challenges, prevalence, and impact of neurological disorders in society

Managing neurological disorders at present is a multifaceted challenge due to the complex nature of the human brain, the diverse spectrum of neurological conditions, and the limitations in current treatments. The brain's complexity complicates efforts to understand the underlying mechanisms of these disorders, deepened by complex neural networks and the presence of the blood–brain barrier^174^. Moreover, many neurological disorders are chronic and progressive, imposing significant emotional, physical, and financial burdens on individuals and society. Enhanced understanding of their etiology is imperative for the development of precise treatments, yet unclear symptoms and social stigma often delay early diagnosis. Furthermore, healthcare differences, limited personalized therapies, and cognitive impairments worsen these conditions. Addressing these complexities necessitates a comprehensive interdisciplinary approach encompassing improved diagnostics, ongoing research, innovative drug delivery methods, increased investment in clinical trials, patient education, integration of mental health services, and global health initiatives. In essence, managing neurological disorders needs a holistic approach to tackle their multifaceted challenges, recognizing their substantial prevalence and impact on society. These disorders encompass a wide range of conditions affecting the nervous system, with conditions like migraines, epilepsy, Alzheimer's, Parkinson's, and multiple sclerosis affecting billions worldwide across various age groups. They entail significant healthcare expenses, including costs related to hospitalization, outpatient care, and medications, while also impacting productivity and contributing to economic burdens. Additionally, these disorders significantly affect the quality of life of those affected and their families, often leading to cognitive decline, physical limitations, and social discrimination. Nevertheless, ongoing research, healthcare interventions, and public health initiatives play a crucial role in addressing these challenges, aiming to reduce the overall societal impact of neurological disorders and improve the well-being of affected individuals^175^.

## 7 Translational medicine options for neurological disorders

The European Society for Translational Medicine (TM) emphasizes its dedication to preventing, diagnosing, and treating worldwide clinical problems. Neurological disorders, impacting around one billion people worldwide, raise major socioeconomic and scientific concerns, particularly with an aging population. Genomic investigations are important because they provide crucial information about disease progression and biomarkers. Despite increasing research into therapeutics for neurological disorders (NDs), the continuance of treatment failures highlights the critical importance of selecting viable targets. Precision medicine, specifically RNA interference therapy and gene therapy, is emerging as a possible treatment option for genetic neuromuscular disorders^176^.

Genomics investigates genes, activities, and genetic variants to understand common disease pathways better. Collaborative initiatives within multinational consortia, such as the PsychENCODE Consortium, improve our ability to anticipate psychiatric symptoms and contribute to a molecular taxonomy for NDs, drawing parallels with oncology breakthroughs^177^.

Technological advances like Comparative Genomic Hybridization array (aCGH) and Next-Generation Sequencing (NGS) enable biomarker development and diagnostic applications. Nonetheless, healthcare structures need to be revised to ensure equitable access to NGS-based diagnosis. Because of the variety of NDDs, which is influenced by genetic and nongenetic variables, a Systems Biology (SB) approach is required. With its diagnostic focus, genomics elucidates pathogenic pathways, aids in patient classification, and discovers novel therapeutic targets, moving Neurology toward more effective therapy^148^. Future challenges include improving diagnostic efficacy, stratifying patients for gene expression-based clinical trials, and finding new therapeutic targets for common disease pathways.

## 8 Neuroimaging modality in assessing oxidative stress in the brain

Neuroimaging and oxidative stress are interconnected domains that are crucial for understanding brain function, and they offer crucial insights into the brain's functioning and susceptibility to various physiological and pathological processes. Neuroimaging techniques like Magnetic resonance imaging (MRI) and computed tomography (CT), produce detailed 3-dimensional images of the inside of the body (PET), and Functional magnetic resonance imaging (fMRI) provides detailed insights into brain structure, blood flow, metabolism, and neural activity. This transformative technology enhances our comprehension of healthy and diseased brains, enabling researchers to detect abnormalities, monitor disease progression, and assess the effectiveness of treatments^178^. Oxidative stress, caused by an imbalance between antioxidants and ROS, can lead to cellular damage and contribute to neurodegenerative disorders such as AD, PD, and MS. The interplay between neuroimaging and oxidative stress is significant in revealing structural and functional brain changes associated with these disorders. For example, neuroimaging can detect hippocampal changes in AD where oxidative stress indicators are elevated, suggesting a link between oxidative damage and neurodegeneration. Research approaches include advanced neuroimaging techniques to assess oxidative stress *in vivo* and correlate imaging data with clinical manifestations^179^.

### 8.1 Advanced neuroimaging techniques for assessing oxidative stress *in vivo*

Oxidative stress is a key factor in many neurological disorders, mainly affecting organs like the brain and mitochondria, which have high oxygen demands. Mitochondrial oxidative respiration generates the most ROS, making the brain highly susceptible to oxidative stress. If untreated, oxidative stress can damage proteins, lipids, and DNA, leading to cell death and contributing to various neurological disorders. Advances in diagnostic techniques, including neuroimaging, have improved the accuracy of diagnosing NDDs and understanding their relationship with oxidative stress^180^. Neuroimaging techniques can assess oxidative stress *in vivo*, with ROS detection and quantification possible through methods like electron paramagnetic resonance (EPR/ESR) and marker probes. Fluorescent probes such as hydrocyanines and BODIPY 581/591 C11 are used to trace different types of ROS and lipid peroxidation despite the challenges posed by the short-lived and low steady-state levels of ROS^181^. Quantifying changes in ROS can be done by measuring the fluorescence ratio from red to green emissions.

### 8.2 Correlation of imaging data with clinical manifestations

Neuroimaging systems have advanced, enabling the diagnosis and treatment of various NDDs by linking brain alterations to clinical symptoms. For instance, in ALS, oxidative stress leads to motor neuron degeneration. The exact cause of ALS is still under investigation, but ROS are implicated, as evidenced by ALS in SOD1 knockout mice^182^. ALS pathology includes motor neuron loss, abnormal mitochondria, and glial activation. Early neuroimaging techniques like SPECT and PET have been used to detect these changes, using tracers to track cerebral metabolism and blood flow.

In AD, neuroimaging tracks amyloid plaques and tau tangles, correlating their accumulation with cognitive decline and memory loss. For psychiatric disorders like schizophrenia and depression, neuroimaging identifies brain changes, aiding in treatment development and efficacy assessment. However, establishing a direct relationship between neuroimaging data and clinical manifestations is challenging due to individual variability^183^, ^184^, ^185^, ^186^. Despite this, correlating imaging data with clinical symptoms can enhance diagnostics and lead to personalized treatments for neurological disorders.

## 9 Synthetic drug and personalized medicine approaches

To address the neurological disorders arising from oxidative stress, researchers are exploring innovative methods like synthetic drugs and personalized therapy. Synthetic medications aim to target specific pathways involved in oxidative stress to mitigate its adverse effects on the neurological system. These drugs may incorporate antioxidants or modulators of enzymes related to oxidative stress, offering a pharmacological approach to rebalance. Personalized medicine utilizes advancements in neuroimaging and molecular profiling to tailor interventions based on an individual's distinct genetic and biochemical characteristics. This approach holds the promise of enhancing treatment outcomes for neurological disorders by identifying specific oxidative stress markers and understanding genetic susceptibilities. The following strategies are primarily undertaken, including synthetic drugs for neurological disorders, potential for personalized medicine in treating NDDs, and individualized treatment strategies based on oxidative stress profiles^187^.

## 10 Synthetic drug and personalized medicine approaches

### 10.1 Synthetic drug for neurological disorder

Synthetic drugs developed for neurological disorders comprise a diverse array of medications aimed at alleviating symptoms or modifying the course of conditions such as AD, PD, epilepsy, MS, and others. These drugs are meticulously designed to address specific disorders, and several categories exist for treating various neurological conditions. Antiepileptic drugs (AEDs), including valproic acid, carbamazepine, and lamotrigine, aim to control seizures in epilepsy by managing neuronal activity^188^. Dopamine agonists like levodopa, pramipexole, and ropinirole mimic dopamine effects in PD to regulate motor symptoms. Cholinesterase inhibitors such as donepezil, rivastigmine, and galantamine slow the breakdown of acetylcholine, a crucial neurotransmitter for memory and cognition in AD. Immunomodulators, exemplified by interferons and glatiramer acetate, modify the immune response in MS, reducing inflammation and slowing disease progression. Muscle relaxants like baclofen and tizanidine are employed to manage muscle spasms and spasticity in conditions such as MS or spinal cord injuries^189^^,^^190^. Selective serotonin reuptake inhibitors (SSRIs) such as fluoxetine, sertraline, and escitalopram, although not specific to neurological disorders, are used in depression and anxiety, conditions often associated with neurological manifestations. Each drug class operates uniquely, targeting particular aspects of neurological conditions to manage symptoms or slow progression, emphasizing the personalized nature of treatment^191^.

### 10.2 Potential for personalized medicine in treating neurodegenerative diseases (NDDs)

Personalized medicine offers a promising approach to NDDs like AD, PD, ALS, and HD, which have complex and varied causes^192^. This approach uses genetic testing to identify specific mutations, facilitating early diagnosis, risk assessment, and individualized treatment strategies. Personalized therapies target particular disease pathways based on a person's genetic and molecular profile, enhancing treatment precision and efficacy^193^. Biomarkers are crucial in early detection, monitoring, and treatment response assessment, allowing for timely adjustments. This approach also guides drug development, focusing on precise molecular targets, and improves clinical trials by stratifying participants based on genetic and molecular profiles. Despite challenges such as cost, accessibility, data privacy, and the complexity of neurological disorders, advancements in genetics and technology continue to advance personalized medicine, offering hope for more effective, tailored treatments^178^.

#### 10.2.1 Individualized treatment strategies based on oxidative stress profiles

Oxidative stress significantly impacts NDDs like AD, PD, and ALS, leading to personalized treatment strategies based on individual oxidative stress profiles. Antioxidant therapy, involving supplements or diets rich in antioxidants (*e*.*g*., vitamins C and E, coenzyme Q10), is tailored to individuals with high oxidative stress^179^. Personalized lifestyle recommendations, including diet, exercise, and stress management, aim to reduce oxidative stress, with exercise plans customized to individual capacities and stress levels. Targeted therapies may involve medications that enhance antioxidant defenses or inhibit excessive free radical production. Regular monitoring of oxidative stress markers allows for personalized therapy adjustments. Combination therapies based on oxidative stress profiles could enhance overall treatment efficacy^179^. However, challenges remain in accurately assessing oxidative stress and translating these assessments into effective treatments, highlighting the need for standardized clinical measurement methods and integrated treatment plans.

## 11 Conclusions

In this review, we tried to explore the intricate roles of ROS in prevalent neurodegenerative conditions, including AD, PD, HD, ALS, and various other diseases. Over the past three decades, intensive investigations have focused on identifying neuropathological, biochemical, and genetic biomarkers during the early phases of these disorders. It is established that oxidative stress undergoes impairment during aging, playing a significant role in this physiological progression. However, uncertainty persists regarding whether oxidative stress can be an early detection marker for age-related dysfunction or a viable therapeutic target.

Elevated levels of ROS have been linked to diverse neurodegenerative conditions, with ROS formation implicated in various disease development pathways, including mitochondrial dysfunction. Despite limited evidence showcasing neuroprotective effects, research studies have yielded positive outcomes. Further investigation is warranted to elucidate specific ROS functions in neurodegenerative disorders and explore novel antioxidant-based treatments. ROS biomarkers hold the potential to be diagnostic tools or therapeutic targets. Antioxidant therapy, incorporating substances like phytochemicals containing dietary supplements alongside moderate physical activity, may mitigate clinical damage induced by oxidative stress.

In conclusion, conclusive evidence demonstrating the neuroprotective potential of antioxidants in mitigating neurodegenerative symptoms remains elusive. Ongoing clinical trials promise favorable outcomes, particularly when antioxidants complement other treatments. Additional research is crucial for delineating ROS roles in various neurodegenerative disorders and formulating antioxidant-based treatment approaches. A deeper understanding of mitochondrial and oxidative stress pathways in aging and neurodegeneration should inspire innovative strategies to enhance the quality of life for the elderly and positively impact society.

## Author contributions

Umesh Chandra Dash: Writing – review & editing, Writing – original draft, Investigation, Conceptualization. Nitish Kumar Bhol: Writing – review & editing, Resources, Conceptualization. Sandeep Kumar Swain: Writing – original draft. Rashmi Rekha Samal: Writing – original draft. Prabhat Kumar Nayak: Writing – original draft. Vishakha Raina: Writing – original draft. Sandeep Kumar Panda: Writing – original draft. Rout George Kerry: Writing – original draft. Asim K. Duttaroy: Writing – review & editing, Conceptualization. Atala Bihari Jena: Writing – review & editing, Writing – original draft, Investigation, Conceptualization.

## Conflicts of interest

The authors declare that there are no conflicts of interest.

## References

[1] Mahalakshmi B., Maurya N., Lee S.D., Bharath Kumar V. (2020). Possible neuroprotective mechanisms of physical exercise in neurodegeneration. Int J Mol Sci.

[2] Huebner E.A., Strittmatter S.M., Koenig E. (2009).

[3] Makkar R., Behl T., Bungau S., Zengin G., Mehta V., Kumar A. (2020). Nutraceuticals in neurological disorders. Int J Mol Sci.

[4] Rehman M.U., Wali A.F., Ahmad A., Shakeel S., Rasool S., Ali R. (2019). Neuroprotective strategies for neurological disorders by natural products: an update. Curr Neuropharmacol.

[5] Uttara B., Singh A., Zamboni P., Mahajan R. (2009). Oxidative stress and neurodegenerative diseases: a review of upstream and downstream antioxidant therapeutic options. Curr Neuropharmacol.

[6] Teleanu D.M., Niculescu A.G., Lungu I.I., Radu C.I., Vladâcenco O., Roza E. (2022). An overview of oxidative stress, neuroinflammation, and neurodegenerative diseases. Int J Mol Sci.

[7] Chandrasekaran A., Idelchik M.D.P.S., Melendez J.A. (2017). Redox control of senescence and age-related disease. Redox Biol.

[8] Moncaster J.A., Pineda R., Moir R.D., Lu S., Burton M.A., Ghosh J.G. (2010). Alzheimer's disease Amyloid-β links lens and brain pathology in Down syndrome. PLoS One.

[9] Lloyd-Price Jason, Lloyd-Price J., Arze C., Ananthakrishnan A.N., Schirmer M., Avila-Pacheco J. (2019). Multi-omics of the gut microbial ecosystem in inflammatory bowel diseases. Nature.

[10] Laberge R.M., Awad P., Campisi J., Desprez P.Y. (2012). Epithelial–mesenchymal transition induced by senescent fibroblasts. Cancer Microenviron.

[11] Antunes Dos Santos A., Ferrer B., Marques Gonçalves F., Tsatsakis A., Renieri E., Skalny A. (2018). Oxidative stress in methylmercury-induced cell toxicity. Toxics.

[12] Mahajan U.V., Varma V.R., Griswold M.E., Blackshear C.T., An Y., Oommen A.M. (2020). Dysregulation of multiple metabolic networks related to brain transmethylation and polyamine pathways in Alzheimer disease: a targeted metabolomic and transcriptomic study. PLoS Med.

[13] Spitz D.R., Azzam E.I., Jian Li J., Gius D. (2004). Metabolic oxidation/reduction reactions and cellular responses to ionizing radiation: a unifying concept in stress response biology. Cancer Metastasis Rev.

[14] Spitz D.R., Hauer-Jensen M. (2014). Ionizing radiation-induced responses: where free radical chemistry meets redox biology and medicine. Antioxid Redox Signal.

[15] Marchitti S.A., Chen Y., Thompson D.C., Vasiliou V. (2011). Ultraviolet radiation: cellular antioxidant response and the role of ocular aldehyde dehydrogenase enzymes. Eye Contact Lens Sci Clin Pract.

[16] Jan A., Azam M., Siddiqui K., Ali A., Choi I., Haq Q. (2015). Heavy metals and human health: mechanistic insight into toxicity and counter defense system of antioxidants. Int J Mol Sci.

[17] Ściskalska M., Zalewska M., Grzelak A., Milnerowicz H. (2014). The influence of the occupational exposure to heavy metals and tobacco smoke on the selected oxidative stress markers in smelters. Biol Trace Elem Res.

[18] Bedard K., Krause K.H. (2007). The nox family of ROS-generating NADPH oxidases: physiology and pathophysiology. Physiol Rev.

[19] Kabe Y., Ando K., Hirao S., Yoshida M., Handa H. (2005). Redox regulation of NF-κB activation: distinct redox regulation between the cytoplasm and the nucleus. Antioxid Redox Signal.

[20] Sena L.A., Chandel N.S. (2012). Physiological roles of mitochondrial reactive oxygen species. Mol Cell.

[21] Battelli M.G., Polito L., Bolognesi A. (2014). Xanthine oxidoreductase in atherosclerosis pathogenesis: not only oxidative stress. Atherosclerosis.

[22] Gandhi S., Abramov A.Y. (2012). Mechanism of oxidative stress in neurodegeneration. Oxid Med Cell Longev.

[23] Salehi I., Zarrinkalam E., Mirzaei F., Abbasi Oshaghi E., Ranjbar K., Asl S.S. (2018). Effects of resistance, endurance, and concurrent exercise on oxidative stress markers and the histological changes of intestine after Morphine withdrawal in rats. Avicenna J Med Biochem.

[24] Elahi B., Elahi B., Chen R. (2009). Effect of transcranial magnetic stimulation on Parkinson motor function—systematic review of controlled clinical trials. Mov Disord.

[25] Taverne Y.J.H.J., Bogers A.J.J.C., Duncker D.J., Merkus D. (2013). Reactive oxygen species and the cardiovascular system. Oxid Med Cell Longev.

[26] Sharifi-Rad M., Anil Kumar N.V., Zucca P., Varoni E.M., Dini L., Panzarini E. (2020). Lifestyle, oxidative stress, and antioxidants: back and forth in the pathophysiology of chronic diseases. Front Physiol.

[27] Glasauer A., Chandel N.S. (2014). Targeting antioxidants for cancer therapy. Biochem Pharmacol.

[28] Li L., Tan J., Miao Y., Lei P., Zhang Q. (2015). Ros and autophagy: interactions and molecular regulatory mechanisms. Cell Mol Neurobiol.

[29] Benfeitas R., Uhlen M., Nielsen J., Mardinoglu A. (2017). New challenges to study heterogeneity in cancer redox metabolism. Front Cell Dev Biol.

[30] Cortat B., Garcia C.C.M., Quinet A., Schuch A.P., De Lima-Bessa K.M., Menck C.F.M. (2013). The relative roles of dna damage induced by UVA irradiation in human cells. Photochem Photobiol Sci.

[31] Salehi A.W., Baglat P., Sharma B.B., Gupta G., Upadhya A. (2020). 2020 International Conference on Smart Electronics and Communication (ICOSEC), Trichy, India.

[32] Tsatsakis A., Tyshko N.V., Docea A.O., Shestakova S.I., Sidorova Y.S., Petrov N.A. (2019). The effect of chronic vitamin deficiency and long term very low dose exposure to 6 pesticides mixture on neurological outcomes – a real-life risk simulation approach. Toxicol Lett.

[33] Reddy P.H. (2014). Increased mitochondrial fission and neuronal dysfunction in Huntington's disease: implications for molecular inhibitors of excessive mitochondrial fission. Drug Discov Today.

[34] Nussbaum R.L. (2017). The identification of alpha-synuclein as the first Parkinson disease gene. J Park Dis.

[35] Salehi B., Calina D., Docea A., Koirala N., Aryal S., Lombardo D. (2020). Curcumin's nanomedicine formulations for therapeutic application in neurological diseases. J Clin Med.

[36] Niedzielska E., Smaga I., Gawlik M., Moniczewski A., Stankowicz P., Pera J. (2016). Oxidative stress in neurodegenerative diseases. Mol Neurobiol.

[37] Finkel T., Holbrook N.J. (2000). Oxidants, oxidative stress and the biology of ageing. Nature.

[38] Payne B.A.I., Chinnery P.F. (2015). Mitochondrial dysfunction in aging: much progress but many unresolved questions. Biochim Biophys Acta BBA - Bioenerg.

[39] Amponsah-Offeh M., Diaba-Nuhoho P., Speier S., Morawietz H. (2023). Oxidative stress, antioxidants and hypertension. Antioxidants.

[40] Godbolt A., DeBoussard C., Stenberg M., Lindgren M., Ulfarsson T., Borg J. (2013). Disorders of consciousness after severe traumatic brain injury: a Swedish-Icelandic study of incidence, outcomes and implications for optimizing care pathways. J Rehabil Med.

[41] Salim S. (2017). Oxidative stress and the central nervous system. J Pharmacol Exp Ther.

[42] Pizzino G., Irrera N., Cucinotta M., Pallio G., Mannino F., Arcoraci V. (2017). Oxidative stress: harms and benefits for human health. Oxid Med Cell Longev.

[43] Williamson M.G., Madureira M., McGuinness W., Heon-Roberts R., Mock E.D., Naidoo K. (2023). Mitochondrial dysfunction and mitophagy defects in *lrrk2-r1441c* Parkinson's disease models. Hum Mol Genet.

[44] Delatycki M.B. (2000). Friedreich ataxia: an overview. J Med Genet.

[45] Delatycki M.B., Corben L.A. (2012). Clinical features of Friedreich Ataxia. J Child Neurol.

[46] Singh A., Kukreti R., Saso L., Kukreti S. (2019). Oxidative stress: a key modulator in neurodegenerative diseases. Molecules.

[47] Shorvon S., Diehl B., Duncan J., Koepp M., Rugg-Gunn F., Sander J. (2016). Neurology.

[48] Osservatorio Regionale per L'Epilessia (OREp) L (1996). ILAE classification of epilepsies: its applicability and practical value of different diagnostic categories. Epilepsia.

[49] Aguiar C.C.T., Almeida A.B., Araújo P.V.P., Abreu R.N.D.C.D., Chaves E.M.C., Vale O.C.D. (2012). Oxidative stress and epilepsy: literature review. Oxid Med Cell Longev.

[50] Borowicz-Reutt K.K., Czuczwar S.J. (2020). Role of oxidative stress in epileptogenesis and potential implications for therapy. Pharmacol Rep.

[51] Tadi P., Lui F., Budd L.A. (2023). StatPearls.

[52] Shao L., Chen S., Ma L. (2022). Secondary brain injury by oxidative stress after cerebral hemorrhage: recent advances. Front Cell Neurosci.

[53] Yao Z., Bai Q., Wang G. (2021). Mechanisms of oxidative stress and therapeutic targets following intracerebral hemorrhage. Oxid Med Cell Longev.

[54] Jinnah H.A., Factor S.A. (2015). Diagnosis and treatment of Dystonia. Neurol Clin.

[55] Quartarone A., Ruge D. (2018). How many types of Dystonia?. Pathophysiological considerations. Front Neurol.

[56] Casper C., Kalliolia E., T. Warner T. (2013). Recent advances in the molecular pathogenesis of Dystonia-plus syndromes and heredodegenerative Dystonias. Curr Neuropharmacol.

[57] Emmady P.D., Schoo C., Tadi, Del Pozo E. (2023). StatPearls.

[58] Wang X., Wang W., Li L., Perry G., Lee H., Zhu X. (2014). Oxidative stress and mitochondrial dysfunction in Alzheimer's disease. Biochim Biophys Acta BBA - Mol Basis Dis.

[59] Tönnies E., Trushina E. (2017). Oxidative stress, synaptic dysfunction, and Alzheimer's disease. J Alzheimers Dis.

[60] Cassidy L., Fernandez F., Johnson J.B., Naiker M., Owoola A.G., Broszczak D.A. (2020). Oxidative stress in Alzheimer's disease: a review on emergent natural polyphenolic therapeutics. Complement Ther Med.

[61] Gella A., Durany N. (2009). Oxidative stress in Alzheimer disease. Cell Adhes Migr.

[62] Huang Z., Chavda V.P., Vora L.K., Gajjar N., Apostolopoulos V., Shah N. (2022). 2-Deoxy-D-Glucose and its derivatives for the COVID-19 treatment: an update. Front Pharmacol.

[63] Tamagno E., Guglielmotto M., Vasciaveo V., Tabaton M. (2021). Oxidative stress and beta amyloid in Alzheimer’s disease. Which comes first: the chicken or the egg?.. Antioxidants.

[64] Su L.J., Zhang J.H., Gomez H., Murugan R., Hong X., Xu D. (2019). Reactive oxygen species-induced lipid peroxidation in apoptosis, autophagy, and ferroptosis. Oxid Med Cell Longev.

[65] Huang M., Jiang X., Liang Y., Liu Q., Chen S., Guo Y. (2017). Berberine improves cognitive impairment by promoting autophagic clearance and inhibiting production of β-amyloid in APP/Tau/PS1 mouse model of Alzheimer's disease. Exp Gerontol.

[66] Swerdlow R.H., Burns J.M., Khan S.M. (2014). The Alzheimer’s disease mitochondrial cascade hypothesis: progress and perspectives. Biochim Biophys Acta.

[67] Nabi S.U., Khan A., Siddiqui E.M., Rehman M.U., Alshahrani S., Arafah A. (2022). Mechanisms of mitochondrial malfunction in Alzheimer's disease: new therapeutic hope. Oxid Med Cell Longev.

[68] Ayala A., Muñoz M.F., Argüelles S. (2014). Lipid peroxidation: production, metabolism, and signaling mechanisms of malondialdehyde and 4-hydroxy-2-nonenal. Oxid Med Cell Longev.

[69] Barrera G., Pizzimenti S., Daga M., Dianzani C., Arcaro A., Cetrangolo G.P. (2018). Lipid peroxidation-derived aldehydes, 4-hydroxynonenal and malondialdehyde in aging-related disorders. Antioxidants.

[70] Sultana R., Perluigi M., Butterfield D.A. (2013). Lipid peroxidation triggers neurodegeneration: a redox proteomics view into the Alzheimer disease brain. Free Radic Biol Med.

[71] Pinho C.M., Teixeira P.F., Glaser E. (2014). Mitochondrial import and degradation of amyloid-β peptide. Biochim Biophys Acta BBA - Bioenerg.

[72] Strope T.A., Wilkins H.M. (2023). Amyloid precursor protein and mitochondria. Curr Opin Neurobiol.

[73] Andronie-Cioara F.L., Ardelean A.I., Nistor-Cseppento C.D., Jurcau A., Jurcau M.C., Pascalau N. (2023). Molecular mechanisms of neuroinflammation in aging and Alzheimer's disease progression. Int J Mol Sci.

[74] Manczak M., Reddy P.H. (2012). Abnormal interaction between the mitochondrial fission protein Drp1 and hyperphosphorylated tau in Alzheimer's disease neurons: implications for mitochondrial dysfunction and neuronal damage. Hum Mol Genet.

[75] Rawat P., Sehar U., Bisht J., Selman A., Culberson J., Reddy P.H. (2022). Phosphorylated tau in Alzheimer's disease and other tauopathies. Int J Mol Sci.

[76] Ramiro-Cortés Y., Morán J. (2009). Role of oxidative stress and JNK pathway in apoptotic death induced by potassium deprivation and staurosporine in cerebellar granule neurons. Neurochem Int.

[77] Kurutas E.B. (2015). The importance of antioxidants which play the role in cellular response against oxidative/nitrosative stress: current state. Nutr J.

[78] Dias V., Junn E., Mouradian M.M. (2013). The role of oxidative stress in Parkinson's disease. J Park Dis.

[79] Hussain G., Wang J., Rasul A., Anwar H., Imran A., Qasim M. (2019). Role of cholesterol and sphingolipids in brain development and neurological diseases. Lipids Health Dis.

[80] Cobb C.A., Cole M.P. (2015). Oxidative and nitrative stress in neurodegeneration. Neurobiol Dis.

[81] Stykel M.G., Ryan S.D. (2022). Nitrosative stress in Parkinson's disease. Npj Park Dis.

[82] Reeve A., Simcox E., Turnbull D. (2014). Ageing and Parkinson’s disease: why is advancing age the biggest risk factor?.. Ageing Res Rev.

[83] Ball N., Teo W.P., Chandra S., Chapman J. (2019). Parkinson's disease and the environment. Front Neurol.

[84] Subramaniam S.R., Chesselet M.F. (2013). Mitochondrial dysfunction and oxidative stress in Parkinson's disease. Prog Neurobiol.

[85] Schon E.A., Przedborski S. (2011). Mitochondria: the next (neurode) generation. Neuron.

[86] Missiroli S., Genovese I., Perrone M., Vezzani B., Vitto V.A.M., Giorgi C. (2020). The role of mitochondria in inflammation: from cancer to neurodegenerative disorders. J Clin Med.

[87] Sampaio T., Savall A., Gutierrez M.Z., Pinton S. (2017). Neurotrophic factors in Alzheimer's and Parkinson's diseases: implications for pathogenesis and therapy. Neural Regen Res.

[88] Soraci L., Gambuzza M.E., Biscetti L., Laganà P., Lo Russo C., Buda A. (2023). Toll-like receptors and NLRP3 inflammasome-dependent pathways in Parkinson's disease: mechanisms and therapeutic implications. J Neurol.

[89] Stefanis L. (2012). Α-synuclein in Parkinson's disease. Cold Spring Harb Perspect Med.

[90] Mahul-Mellier A.L., Burtscher J., Maharjan N., Weerens L., Croisier M., Kuttler F. (2020). The process of lewy body formation, rather than simply α-synuclein fibrillization, is one of the major drivers of neurodegeneration. Proc Natl Acad Sci.

[91] Chen X., Guo C., Kong J. (2012). Oxidative stress in neurodegenerative diseases. Neural Regen Res.

[92] Sayre L.M., Perry G., Smith M.A. (2008). Oxidative stress and neurotoxicity. Chem Res Toxicol.

[93] Olufunmilayo E.O., Gerke-Duncan M.B., Holsinger R.M.D. (2023). Oxidative stress and antioxidants in neurodegenerative disorders. Antioxidants.

[94] Zheng J., Winderickx J., Franssens V., Liu B. (2018). A mitochondria-associated oxidative stress perspective on Huntington's disease. Front Mol Neurosci.

[95] Reddy N.M., Qureshi W., Potteti H., Kalvakolanu D.V., Reddy S.P., Natarajan V., Parinandi N.L. (2014).

[96] Bordoni L., Gabbianelli R. (2020). Mitochondrial dna DNA and neurodegeneration: any role for dietary antioxidants?.. Antioxidants.

[97] Filograna R., Mennuni M., Alsina D., Larsson N. (2021). Mitochondrial DNA copy number in human disease: the more the better?.. FEBS Lett.

[98] Bossy-Wetzel E., Petrilli A., Knott A.B. (2008). Mutant huntingtin and mitochondrial dysfunction. Trends Neurosci.

[99] Ebanks B., Chakrabarti L. (2022). Mitochondrial ATP synthase is a target of oxidative stress in neurodegenerative diseases. Front Mol Biosci.

[100] Sorolla M.A., Rodríguez-Colman M.J., Tamarit J., Ortega Z., Lucas J.J., Ferrer I. (2010). Protein oxidation in Huntington disease affects energy production and vitamin B6 metabolism. Free Radic Biol Med.

[101] Franco-Iborra S., Vila M., Perier C. (2018). Mitochondrial quality control in neurodegenerative diseases: focus on Parkinson's disease and Huntington's disease. Front Neurosci.

[102] Gray M., Shirasaki D.I., Cepeda C., Andre V.M., Wilburn B., Lu X.-H. (2008). Full-length human mutant huntingtin with a stable polyglutamine repeat can elicit progressive and selective neuropathogenesis in bachd mice. J Neurosci.

[103] Joviano-Santos J.V., Santos-Miranda A., Botelho A.F.M., De Jesus I.C.G., Andrade J.N., De Oliveira Barreto T. (2019). Increased oxidative stress and Camkii activity contribute to electro-mechanical defects in cardiomyocytes from a murine model of Huntington's disease. FEBS J.

[104] Hassan W., Noreen H., Rehman S., Kamal M.A., Da Rocha J.B.T. (2022). Association of Oxidative stress with neurological disorders. Curr Neuropharmacol.

[105] Korovesis D., Rubio-Tomás T., Tavernarakis N. (2023). Oxidative stress in age-related neurodegenerative diseases: an overview of recent tools and findings. Antioxidants.

[106] Tang Q., Liu H., Shi X.J., Cheng Y. (2020). Blood oxidative stress marker aberrations in patients with Huntington's disease: a Meta-Analysis Study. Oxid Med Cell Longev.

[107] Fraunberger E.A., Scola G., Laliberté V.L.M., Duong A., Andreazza A.C. (2016). Redox modulations, antioxidants, and neuropsychiatric disorders. Oxid Med Cell Longev.

[108] Smith E.F., Shaw P.J., De Vos K.J. (2019). The role of mitochondria in Amyotrophic lateral sclerosis. Neurosci Lett.

[109] Bonnefont-Rousselot D., Lacomblez L., Jaudon M.C., Lepage S., Salachas F., Bensimon G. (2000). Blood oxidative stress in amyotrophic lateral sclerosis. J Neurol Sci.

[110] Muyderman H., Chen T. (2014). Mitochondrial dysfunction in Amyotrophic lateral sclerosis – a valid pharmacological target?.. Br J Pharmacol.

[111] Sidorova Y., Domanskyi A. (2020). Detecting oxidative stress biomarkers in neurodegenerative disease models and patients. Methods Protoc.

[112] Mitsumoto H., Garofalo D.C., Santella R.M., Sorenson E.J., Oskarsson B., Fernandes J.A.M. (2020). Plasma creatinine and oxidative stress biomarkers in Amyotrophic lateral sclerosis. Amyotroph Lateral Scler Front Degener.

[113] Ilieva E.V., Ayala V., Jove M., Dalfo E., Cacabelos D., Povedano M. (2007). Oxidative and endoplasmic reticulum stress interplay in sporadic Amyotrophic lateral sclerosis. Brain.

[114] Cunha-Oliveira T., Montezinho L., Mendes C., Firuzi O., Saso L., Oliveira P.J. (2020). Oxidative stress in amyotrophic lateral sclerosis: pathophysiology and opportunities for pharmacological intervention. Oxid Med Cell Longev.

[115] Barber S.C., Mead R.J., Shaw P.J. (2006). Oxidative stress in als: a mechanism of neurodegeneration and a therapeutic target. Biochim Biophys Acta BBA - Mol Basis Dis.

[116] Wendimu M.Y., Hooks S.B. (2022). Microglia phenotypes in aging and neurodegenerative diseases. Cells.

[117] Muzio L., Viotti A., Martino G. (2021). Microglia in neuroinflammation and neurodegeneration: from understanding to therapy. Front Neurosci.

[118] Orth M., Schapira A.H.V. (2001). Mitochondria and degenerative disorders. Am J Med Genet.

[119] Lin M.T., Beal M.F. (2006). Mitochondrial dysfunction and oxidative stress in neurodegenerative diseases. Nature.

[120] Das A., McDowell M., O'Dell C.M., Busch M.E., Smith J.A., Ray S.K. (2010). Post-treatment with voltage-gated Na^+^ channel blocker attenuates kainic acid-induced apoptosis in rat primary hippocampal neurons. Neurochem Res.

[121] Hroudová J., Singh N., Fišar Z. (2014). Mitochondrial dysfunctions in neurodegenerative diseases: relevance to Alzheimer's disease. Biomed Res Int.

[122] Moran P., Coffey C., Romaniuk H., Olsson C., Borschmann R., Carlin J.B. (2012). The natural history of self-harm from adolescence to young adulthood: a population-based cohort study. Lancet.

[123] Musatov A., Robinson N.C. (2012). Susceptibility of mitochondrial electron-transport complexes to oxidative damage. Focus on cytochrome c oxidase. Free Radic Res.

[124] Dehay B., Ramirez A., Martinez-Vicente M., Perier C., Canron M.-H., Doudnikoff E. (2012). Loss of p-type atpase atp13a2/park9 function induces general lysosomal deficiency and leads to Parkinson disease neurodegeneration. Proc Natl Acad Sci.

[125] Wu B.-S., Zhang Y.-R., Li H.-Q., Kuo K., Chen S.D., Dong Q. (2021). Cortical structure and the risk for Alzheimer's disease: a bidirectional Mendelian randomization study. Transl Psychiatry.

[126] Aronis A., Melendez J.A., Golan O., Shilo S., Dicter N., Tirosh O. (2003). Potentiation of Fas-mediated apoptosis by attenuated production of mitochondria-derived reactive oxygen species. Cell Death Differ.

[127] Manczak M., Calkins M.J., Reddy P.H. (2011). Impaired mitochondrial dynamics and abnormal interaction of amyloid beta with mitochondrial protein Drp1 in neurons from patients with Alzheimer's disease: implications for neuronal damage. Hum Mol Genet.

[128] Butt A., Verkhratsky A. (2018). Neuroglia: realising their true potential. Brain Neurosci Adv.

[129] Rodríguez-Gómez J.A., Kavanagh E., Engskog-Vlachos P., Engskog M.K.R., Herrera A.J., Espinosa-Oliva A.M. (2020). Microglia: agents of the cns pro-inflammatory response. Cells.

[130] Miao J., Ma H., Yang Y., Liao Y., Lin C., Zheng J. (2023). Microglia in Alzheimer's disease: pathogenesis, mechanisms, and therapeutic potentials. Front Aging Neurosci.

[131] Lee C.Y.D., Landreth G.E. (2010). The role of microglia in amyloid clearance from the AD brain. J Neural Transm.

[132] Liang T., Zhang Y., Wu S., Chen Q., Wang L. (2022). The role of nlrp3 inflammasome in Alzheimer's disease and potential therapeutic targets. Front Pharmacol.

[133] Chen K., Wang H., Ilyas I., Mahmood A., Hou L. (2023). Microglia and astrocytes dysfunction and key neuroinflammation-based biomarkers in Parkinson's disease. Brain Sci.

[134] Palpagama T.H., Waldvogel H.J., Faull R.L.M., Kwakowsky A. (2019). The role of microglia and astrocytes in Huntington's disease. Front Mol Neurosci.

[135] Palpagama T., Mills A.R., Ferguson M.W., Vikas Ankeal P., Turner C., Tippett L. (2023). Microglial and astrocytic responses in the human midcingulate cortex in Huntington's disease. Ann Neurol.

[136] Simpson D.S.A., Oliver P.L. (2020). Ros generation in microglia: understanding oxidative stress and inflammation in neurodegenerative disease. Antioxidants.

[137] Koppula S., Kumar H., Kim I.S., Choi D.K. (2012). Reactive oxygen species and inhibitors of inflammatory enzymes, NADPH oxidase, and inos in experimental models of Parkinson's disease. Mediators Inflamm.

[138] Han S., Gim Y., Jang E.H., Hur E.M. (2022). Functions and dysfunctions of oligodendrocytes in neurodegenerative diseases. Front Cell Neurosci.

[139] Qin J., Ma Z., Chen X., Shu S. (2023). Microglia activation in central nervous system disorders: a review of recent mechanistic investigations and development efforts. Front Neurol.

[140] Deneubourg C., Ramm M., Smith L.J., Baron O., Singh K., Byrne S.C. (2022). The spectrum of neurodevelopmental, neuromuscular and neurodegenerative disorders due to defective autophagy. Autophagy.

[141] Jiang M., He J., Sun Y., Dong X., Yao J., Gu H. (2021). Leptin induced tlr4 expression *via* the JAK2–STAT3 pathway in obesity-related osteoarthritis. Oxid Med Cell Longev.

[142] Shao R., Sun D., Hu Y., Cui D. (2021). White matter injury in the neonatal hypoxic-ischemic brain and potential therapies targeting microglia. J Neurosci Res.

[143] Zhao H., Wu L., Yan G., Chen Y., Zhou M., Wu Y. (2021). Inflammation and tumor progression: signaling pathways and targeted intervention. Signal Transduct Target Ther.

[144] Phatnani H., Maniatis T. (2015). Astrocytes in neurodegenerative disease: table 1. Cold Spring Harb Perspect Biol.

[145] Nutma E., Van Gent D., Amor S., Peferoen L.A.N. (2020). Astrocyte and oligodendrocyte cross-talk in the central nervous system. Cells.

[146] Tan R., Hong R., Sui C., Yang D., Tian H., Zhu T. (2023). The role and potential therapeutic targets of astrocytes in central nervous system demyelinating diseases. Front Cell Neurosci.

[147] Singh S., Singh T.G. (2020). Role of nuclear factor kappa b (NF-*κ*B) signalling in neurodegenerative diseases: an mechanistic approach. Curr Neuropharmacol.

[148] Sun H., Shen X.-R., Fang Z.B., Jiang Z.Z., Wei X.J., Wang Z.Y. (2021). Next-generation sequencing technologies and neurogenetic diseases. Life.

[149] Khoshnan A., Sabbaugh A., Calamini B., Marinero S.A., Dunn D.E., Yoo J.H. (2017). IKKΒ and mutant huntingtin interactions regulate the expression of il-34: implications for microglial-mediated neurodegeneration in hd. Hum Mol Genet.

[150] Tanaka M., Tóth F., Polyák H., Szabó Á., Mándi Y., Vécsei L. (2021). Immune Influencers in Action: metabolites and enzymes of the tryptophan-kynurenine metabolic pathway. Biomedicines.

[151] Dantzer R., Dantzer R., Capuron L. (2017). Inflammation-associated depression: evidence, mechanisms and implications.

[152] Orsini F., De Blasio D., Zangari R., Zanier E.R., De Simoni M.-G. (2014). Versatility of the complement system in neuroinflammation, neurodegeneration and brain homeostasis. Front Cell Neurosci.

[153] Islam F., Roy S., Zehravi M., Paul S., Sutradhar H., Yaidikar L. (2023). Polyphenols targeting map kinase signaling pathway in neurological diseases: understanding molecular mechanisms and therapeutic targets. Mol Neurobiol.

[154] Moens U., Kostenko S., Sveinbjørnsson B. (2013). The role of mitogen-activated protein kinase-activated protein kinases (mapkapks) in inflammation. Genes.

[155] Deming Y., Li Z., Benitez B.A., Cruchaga C. (2018). Triggering receptor expressed on myeloid cells 2 (TREM2): a potential therapeutic target for Alzheimer disease?.. Expert Opin Ther Targets.

[156] Gaschler M.M., Stockwell B.R. (2017). Lipid peroxidation in cell death. Biochem Biophys Res Commun.

[157] Wojtunik-Kulesza K.A., Oniszczuk A., Oniszczuk T., Waksmundzka-Hajnos M. (2016). The influence of common free radicals and antioxidants on development of Alzheimer's disease. Biomed Pharmacother.

[158] Gholamian-Dehkordi N., Luther T., Asadi-Samani M., Mahmoudian-Sani M.R. (2017). An overview on natural antioxidants for oxidative stress reduction in cancers; a systematic review. Immunopathol Persa.

[159] Huang K., Chen Y., Zhang R., Wu Y., Ma Y., Fang X. (2018). Honokiol induces apoptosis and autophagy *via* the ROS/ERK1/2 signaling pathway in human osteosarcoma cells *in vitro* and *in vivo*. Cell Death Dis.

[160] Lohan S.B., Vitt K., Scholz P., Keck C.M., Meinke M.C. (2018). ROS production and glutathione response in keratinocytes after application of β-carotene and VIS/NIR irradiation. Chem Biol Interact.

[161] Tu W., Wang H., Li S., Liu Q., Sha H. (2019). The anti-inflammatory and anti-oxidant mechanisms of the Keap1/Nrf2/ARE signaling pathway in chronic diseases. Aging Dis.

[162] Limón-Pacheco J.H., Hernández N.A., Fanjul-Moles M.L., Gonsebatt M.E. (2007). Glutathione depletion activates mitogen-activated protein kinase (MAPK) pathways that display organ-specific responses and brain protection in mice. Free Radic Biol Med.

[163] Moldogazieva N.T., Mokhosoev I.M., Feldman N.B., Lutsenko S.V. (2018). ROS and RNS signalling: adaptive redox switches through oxidative/nitrosative protein modifications. Free Radic Res.

[164] Ji L.L., Gomez-Cabrera M., Vina J. (2006). Exercise and Hormesis: activation of cellular antioxidant signaling pathway. Ann N Y Acad Sci.

[165] Yamamoto M., Yamaguchi T., Yamauchi M., Yano S., Sugimoto T. (2008). Serum pentosidine levels are positively associated with the presence of vertebral fractures in postmenopausal women with type 2 diabetes. J Clin Endocrinol Metab.

[166] De Oliveira M.R., De Souza I.C.C., Brasil F.B. (2021). Promotion of mitochondrial protection by emodin in methylglyoxal-treated human neuroblastoma SH-SY5Y cells: involvement of the ampk/nrf2/ho-1 axis. Neurotox Res.

[167] Ugarte A., Corbacho D., Aymerich M.S., García-Osta A., Cuadrado-Tejedor M., Oyarzabal J. (2018). Impact of neurodegenerative diseases on drug binding to brain tissues: from animal models to human samples. Neurotherapeutics.

[168] Tan B.L., Norhaizan M.E., Liew W.P.P. (2018). Nutrients and oxidative stress: Friend or Foe?.. Oxid Med Cell Longev.

[169] Bellizzi M.C., Franklin M.F., Duthie G.G., James W.P. (1994). Vitamin E and coronary heart disease: the European paradox. Eur J Clin Nutr.

[170] Gey K.F., Puska P. (1989). Plasma vitamins E and A inversely correlated to mortality from ischemic heart disease in cross-cultural epidemiology. Ann N Y Acad Sci.

[171] Rimm E.B., Stampfer M.J., Ascherio A., Giovannucci E., Colditz G.A., Willett W.C. (1993). Vitamin E consumption and the risk of coronary heart disease in men. N Engl J Med.

[172] Oyewole A.O., Birch-Machin M.A. (2015). Mitochondria-targeted antioxidants. FASEB J.

[173] Jain K.K., Jain K.K. (2021). Textbook of personalized medicine.

[174] Blanchard J.W., Victor M.B., Tsai L.H. (2022). Dissecting the complexities of Alzheimer disease with *in vitro* models of the human brain. Nat Rev Neurol.

[175] Shidhaye R., Lund C., Chisholm D. (2015). Closing the treatment gap for mental, neurological and substance use disorders by strengthening existing health care platforms: strategies for delivery and integration of evidence-based interventions. Int J Ment Health Syst.

[176] Gentile G., Cavallaro S. (2019). Translational medicine in neurological disorders: a genomic perspective. Curr Genomics.

[177] Geschwind D.H., Flint J. (2015). Genetics and genomics of psychiatric disease. Science.

[178] Rose N. (2013). Personalized medicine: promises, problems and perils of a new paradigm for healthcare. J Peking Univ.

[179] Morén C., deSouza R.M., Giraldo D.M., Uff C. (2022). Antioxidant therapeutic strategies in neurodegenerative diseases. Int J Mol Sci.

[180] Cenini G., Lloret A., Cascella R. (2020). Oxidative stress and mitochondrial damage in neurodegenerative diseases: from molecular mechanisms to targeted therapies. Oxid Med Cell Longev.

[181] Murphy M.P., Bayir H., Belousov V., Chang C.J., Davies K.J.A., Davies M.J. (2022). Guidelines for measuring reactive oxygen species and oxidative damage in cells and *in vivo*. Nat Metab.

[182] Davenport F., Gallacher J., Kourtzi Z., Koychev I., Matthews P.M., Oxtoby N.P. (2022). Neurodegenerative disease of the brain: a survey of interdisciplinary approaches. J R Soc Interface.

[183] Ismail R., Parbo P., Madsen L.S., Hansen A.K., Hansen K.V., Schaldemose J.L. (2020). The relationships between neuroinflammation, beta-amyloid and tau deposition in Alzheimer’s disease: a longitudinal PET study. J Neuroinflammation.

[184] Saberi S., Stauffer J.E., Schulte D.J., Ravits J. (2015). Neuropathology of amyotrophic lateral sclerosis and its variants. Neurol Clin.

[185] Bai L., Ai L., Ding M., He Y., Lao L., Liang F. (2014). Imaging neurodegenerative diseases: mechanisms and interventions. Biomed Res Int.

[186] Zhang H., Wei W., Zhao M., Ma L., Jiang X., Pei H. (2021). Interaction between Aβ and tau in the pathogenesis of Alzheimer’s disease. Int J Biol Sci.

[187] Singh A., Kukreti R., Saso L., Kukreti S. (2019). Oxidative stress: a key modulator in neurodegenerative diseases. Mol Basel Switz.

[188] Naqvi S., Panghal A., Flora S.J.S. (2020). Nanotechnology: a promising approach for delivery of neuroprotective drugs. Front Neurosci.

[189] Clout A.E., Della Pasqua O., Hanna M.G., Orlu M., Pitceathly R.D.S. (2019). Drug repurposing in neurological diseases: an integrated approach to reduce trial and error. J Neurol Neurosurg Psychiatry.

[190] Khatri D.K., Kadbhane A., Patel M., Nene S., Atmakuri S., Srivastava S. (2021). Gauging the role and impact of drug interactions and repurposing in neurodegenerative disorders. Curr Res Pharmacol Drug Discov.

[191] Edinoff A.N., Akuly H.A., Hanna T.A., Ochoa C.O., Patti S.J., Ghaffar Y.A. (2021). Selective serotonin reuptake inhibitors and adverse effects: a narrative review. Neurol Int.

[192] Ciurea A.V., Mohan A.G., Covache-Busuioc R.A., Costin H.P., Glavan L.A., Corlatescu A.D. (2023). Unraveling molecular and genetic insights into neurodegenerative diseases: advances in understanding Alzheimer's, Parkinson's, and Huntington's diseases and amyotrophic lateral sclerosis. Int J Mol Sci.

[193] Hensing T., Chawla A., Batra R., Salgia R., Maltsev N., Rzhetsky A., Gilliam T.C. (2014).

